# The Association between Quality of HIV Care, Loss to Follow-Up and Mortality in Pediatric and Adolescent Patients Receiving Antiretroviral Therapy in Nigeria

**DOI:** 10.1371/journal.pone.0100039

**Published:** 2014-07-30

**Authors:** Bisola Ojikutu, Molly Higgins-Biddle, Dana Greeson, Benjamin R. Phelps, Anouk Amzel, Emeka Okechukwu, Usman Kolapo, Howard Cabral, Ellen Cooper, Lisa R. Hirschhorn

**Affiliations:** 1 John Snow Inc., Boston, Massachusetts, United States of America; 2 Massachusetts General Hospital, Infectious Disease Division, Boston, Massachusetts, United States of America; 3 Columbia University, Department of Epidemiology, New York, New York, United States of America; 4 United States Agency for International Development (USAID), Washington, D. C., United States of America; 5 United States Agency for International Development (USAID), Abuja, Nigeria; 6 Indepth Precision, Abuja, Nigeria; 7 Boston University School of Public Health, Department of Biostatistics, Boston, Massachusetts, United States of America; 8 Boston University School of Medicine, Boston, Massachusetts, United States of America; 9 Harvard Medical School, Department of Global Health and Social Medicine, Harvard Medical School, Boston, Massachusetts, United States of America; University of São Paulo School of Medicine, Brazil

## Abstract

Access to pediatric HIV treatment in resource-limited settings has risen significantly. However, little is known about the quality of care that pediatric or adolescent patients receive. The objective of this study is to explore quality of HIV care and treatment in Nigeria and to determine the association between quality of care, loss-to-follow-up and mortality. A retrospective cohort study was conducted including patients ≤18 years of age who initiated ART between November 2002 and December 2011 at 23 sites across 10 states. 1,516 patients were included. A quality score comprised of 6 process indicators was calculated for each patient. More than half of patients (55.5%) were found to have a high quality score, using the median score as the cut-off. Most patients were screened for tuberculosis at entry into care (81.3%), had adherence measurement and counseling at their last visit (88.7% and 89.7% respectively), and were prescribed co-trimoxazole at some point during enrollment in care (98.8%). Thirty-seven percent received a CD4 count in the six months prior to chart review. Mortality within 90 days of ART initiation was 1.9%. A total of 4.2% of patients died during the period of follow-up (mean: 27 months) with 19.0% lost to follow-up. In multivariate regression analyses, weight for age z-score (Adjusted Hazard Ratio (AHR): 0.90; 95% CI: 0.85, 0.95) and high quality indicator score (compared a low score, AHR: 0.43; 95% CI: 0.26, 0.73) had a protective effect on mortality. Patients with a high quality score were less likely to be lost to follow-up (Adjusted Odds Ratio (AOR): 0.42; 95% CI: 0.32, 0.56), compared to those with low score. These findings indicate that providing high quality care to children and adolescents living with HIV is important to improve outcomes, including lowering loss to follow-up and decreasing mortality in this age group.

## Introduction

Though numerous challenges have limited scale-up of pediatric antiretroviral therapy (ART), significant progress has been made. [1]–[3] Access to pediatric ART in resource limited settings has risen more than 7-fold from 75,000 children receiving ART in low and middle income countries in 2005 to 562,000 by the end of 2011. [4] As pediatric treatment becomes more widely available, determining standards for and measuring the quality of HIV care and treatment that children receive has become a high priority. In order to derive the full benefit of ART, children and adolescents must be provided with high quality care and treatment that addresses their multifaceted needs, including adherence counseling, disclosure support, laboratory monitoring, and opportunistic infection screening along with other critical services.

The Institute of Medicine defines health care quality as the extent to which health services provided to individuals and populations improve desired health outcomes and are consistent with current professional knowledge. [5] Performance indicators are measurement tools that may be used to assess health care quality. [6] These indicators fall into three categories: (1) structure (characteristics of the health care setting such as human resource availability); (2) process (aspects of the encounter with the patient such as which tests are ordered); or (3) outcome (the patient's subsequent health status). [7]–[10] Structure and process may influence outcome, indirectly or directly. [11] Understanding this relationship is essential for quality improvement efforts, particularly in settings where resources to institute structural or procedural change are limited. Numerous studies conducted in sub-Saharan Africa have assessed the impact of selected structural changes, such as task-shifting and decentralization of services, on HIV treatment outcomes. [12]–[17] Process indicators capturing key services, such as semi-annual CD4 count monitoring, routine opportunistic infection screening and regular adherence support, are frequently collected for programmatic monitoring and evaluation. However, the correlation between process indicators such as these and clinical outcomes of HIV care and treatment has not been well explored in resource limited settings. [18], [19]

Moreover, quality assessment of pediatric HIV care and treatment lags far behind that of adults internationally. Several national programs have identified process and outcomes indicators to guide improvement efforts. However, few reports of HIV care quality that focus on pediatric or adolescent services have been published. [20]–[22] Reports describing clinical outcomes in this age group have documented significant challenges, with numerous studies noting high rates of loss to follow-up and early mortality. [23]–[28] Many factors—including late presentation, malnutrition, lack of caregiver involvement, nondisclosure, and HIV-related stigma—contribute to these findings. [27], [29], [30] Whether the quality of care received by children and young adults is associated with loss to follow-up or mortality has not been assessed. As pediatric and adolescent ART becomes more widely available globally, identifying appropriate measures of quality and determining their association with outcomes is critical and should help guide future programmatic planning.

Nigeria is home to the second largest number of people living with HIV in the world after South Africa. In 2012, approximately 3,400,000 people in Nigeria were living with HIV, including 430,000 children. [31] Though coordinated efforts have been underway in Nigeria to increase access to ART for pediatric patients since 2005, a significant disparity exists between pediatric and adult ART coverage (7% versus 26%, respectively). [32] Limited data exist describing clinical outcomes or quality of care in pediatric or adolescent patients on ART in Nigeria. [33], [34] The objective of this study is to explore quality of care received by pediatric and adolescent patients receiving ART in Nigeria and to determine the association between quality of care and loss to follow-up and mortality.

## Methods

### Ethical Review

The study protocol and assessment tools were submitted to the National Health Research Ethics Committee of Nigeria (NHREC) and approved on April 9th, 2011. Individual signed, written informed consent from participants was waived by NHREC. Patient information was de-identified prior to analysis. Unique patient identifiers were assigned to each patient to protect patient confidentiality.

### Study design

A retrospective cohort study was conducted including patients enrolled in care between November 2002 and December 2011. This chart review was a component of a larger assessment of access to pediatric and adolescent treatment services funded by the United States Government/PEPFAR Nigeria Program. [File S1] Purposive sampling was used to select 23 sites providing antiretroviral therapy from 10 states across the 6 geopolitical zones of Nigeria. At the time of this assessment, all pediatric HIV care and treatment in Nigeria was provided at hospitals that offered secondary and tertiary-level specialty services. The criteria guiding site selection included geographic location and setting (i.e., urban, peri-urban, or rural). Safety concerns limited inclusion of sites in certain states. All treatment sites are monitored by the Federal Ministry of Health and supported by a variety of US government funded implementing partners (IPs).

Charts representing 10% of the total number of patients 0–18 years of age receiving antiretroviral therapy (ART) in the 10 states chosen were selected using random sampling. Each site was asked to provide a list of all enrolled patients 0–18 years of age meeting the inclusion criteria by medical record number. Chart design and contents were standardized in 2008 to include the same variables across all sites throughout the country, and sites maintained either paper or electronic records.

Patients were eligible for inclusion in the study if they were 0–18 years of age and initiated ART during the study period. Follow-up was censored either at the time of the loss to follow-up (as defined below), death or the end of the study period.

### Outcomes

The two main outcomes were lost to follow-up and death. Loss to follow-up was defined as no evidence of a visit to the clinic or drug pick up for 90 days following the last scheduled appointment as documented in the chart. Death was only counted if verified by the patient's family or if death occurred within the hospital.

### Independent variables including quality of care indicators

The primary independent variable of interest was a quality score comprised of six process indicators: (1) screening for tuberculosis at entry into care, (2) adherence measurement at last visit, (3) adherence counseling at last visit, (4) prescription of co-trimoxazole at any time since enrollment, (5) at least one CD4 count in the last six months, and (6) documented weight at last visit. These indicators are recommended by the World Health Organization and should be standard components of clinical practice as outlined by the 2005 and 2010 National Guidelines on Paediatric HIV and AIDS Treatment and Care in Nigeria. [35], [36] Recommendations regarding these process indicators did not change over time. Similar indicators have been used to assess pediatric HIV care and treatment quality in other studies conducted in resource limited settings. [22] Screening for tuberculosis in pediatric and adolescent patients includes symptom assessment (poor weight gain, fever, and cough), determination of contact history with a known TB case, clinical examination and radiology followed by sputum induction, if warranted. The standard procedure for adherence assessment in HIV clinics in Nigeria is to ask the patient or the caregiver the number of ART doses missed within the last three days. Review of pharmacy records, returned syrup measurement and pill counting are not routinely conducted. Adherence counseling should be offered at every visit. More intensive counseling is provided if the patient or caregiver reports missed ART doses. CD4 count testing and co-trimoxazole are provided free of charge at all sites. A quality score was calculated with one point assigned for each service received and zero points assigned if the service was not received, for a total of six points. For ease of interpretation, the score was categorized into “high quality” versus “low quality” using the median score as a cutoff for bivariate and multivariate regression models.

Additional patient characteristics and clinical variables related to the outcomes of interest were also collected including: gender, age at ART initiation, CD4 count/percentage (baseline and most recent value), baseline weight/height (to determine age-for-weight z-score), current ART regimen, and facility type (rural, peri-urban and urban). Viral load measurement was not standard of care during the study period, and therefore was not included as an outcome measure.

Baseline immunosuppression at entry into care was calculated using patient age and either initial CD4 count or initial CD4 percentage. For patients less than two years of age, “severe immunosuppression” was defined as an initial CD4 count less than 750 cells/mm^3^ or a percentage less than 15%, while “moderate immunosuppression” was defined as CD4 count between 750 and 1500 cells/mm^3^ or a percentage of between 15% and 25%. “No immunosuppression” was defined as an initial CD4 count of 1500 cells/mm^3^ or more or a percentage of 25% or more. For patients between two and five years of age, “severe immunosuppression” was defined as an initial CD4 count less than 500 cells/mm^3^ or percentage less than 15%, “moderate immunosuppression” as an initial CD4 count of between 500 and 1000 cells/mm^3^ or percentage of between 15% and 25%, and “no immunosuppression” as an initial CD4 count of 1000 cells/mm^3^ or more or percentage of 25% or more. For patients five years of age or older, “severe immunosuppression” was defined as an initial CD4 count less than 200 cells/mm^3^ or percentage less than 15%, “moderate immunosuppression” as an initial CD4 count of between 200 and 500 cells/mm^3^ or percentage of between 15% and 25%, and “no immunosuppression” as an initial CD4 count of 500 cells/mm^3^ or more or percentage of 25% or more. [35]

### Data Analysis

Means and standard deviations were computed for continuous variables and counts with percentages for categorical variables. Differences in mortality and loss to follow-up by age group and differences in quality indicators by year of initial visit were compared using chi-square statistics. Bivariate methods were used to examine the relationships of individual independent variables with the primary outcomes of survival and loss-to-follow up, including Kaplan-Meier estimation with log rank testing. One predictor Cox proportional hazards regression models were used for survival and one predictor logistic regression models for loss to follow-up.

In survival analyses, associations were estimated using hazard ratios (HR) with 95 percent confidence intervals (CI). In the analysis of loss to follow-up, associations were estimated using odds ratios (OR) with 95 percent CIs. The multivariate logistic regression models included independent variables that were significant in the bivariate models defined as p<0.05 and/or were potential confounders of the relationship between the quality score and the outcomes (e.g., weight for age (z-score) and age at ART initiation). The multivariate Cox regression model was limited to four predictors due to the number of deaths in the sample, using a guide of ten events or deaths required per predictor. [37]

The proportional hazards assumption was checked by graphical methods and by testing interaction terms that included the log of follow-up time. The discrimination ability of the logistic models was measured by c-statistics with calibration assessed using Hosmer-Lemeshow chi-square statistics and their associated p-values. Where data were sufficient, we tested for interactions among the independent variables in these models. Patient-level variability across sites was investigated using the intra-cluster correlation coefficient (ICC). The ICC was close to zero, indicating similarity between within-site variability and variability across sites. We employed an alpha of 0.05 in all statistical tests to determine statistical significance. All data management and statistical analyses were performed using SAS for Windows version 9.2.

## Results

### Study population

A total of 1,516 patients were sampled from 23 sites. Most patients (73.6%) received care at urban facilities (Table 1). The average human resource distribution for pediatric HIV care and treatment was similar across facilities with an average of 23 full-time clinical staff (including doctors, nurses, pharmacists and counselors) per site. Approximately one-quarter of patients were 24 months old or younger at ART initiation, while 40.0% were between two and six years old, 21.1% were six to nine years old, and 14.9% were 10 to 18 years old (Table 1). Most patients had an initial visit and initiated ART between 2008 and 2011. Approximately one-half of patients were male (52.8%). Almost half of patients were severely immunosuppressed at baseline (46.3%), 32.5% were moderately immunosuppressed, and 21.3% were not immunosuppressed. The mean weight for age (z-score) at baseline was −1.08 (±4.04).

**Table 1:** Characteristics of sampled pediatric and adolescent patients (age 0 to 18 years).

	*All Patients (n = 1516)*	
	N	Mean (SD) or %
**Demographics**		
Age at ART initiation		
0–24 months	363	24.0%
25–71 months	605	40.0%
6–9 years	318	21.1%
10–18 years	225	14.9%
Gender (Male)	799	52.8%
**Clinical factors**		
Baseline immunosuppression^1^		
Severe	666	46.3%
0–24 months	165	24.8%
25–71 months	256	38.4%
6–9 years	124	18.6%
10–18 years	121	18.2%
Moderate	468	32.5%
0–24 months	106	22.7%
25–71 months	202	43.3%
6–9 years	92	19.7%
10–18 years	67	14.4%
No suppression	306	21.3%
0–24 months	47	15.4%
25–71 months	126	41.3%
6–9 years	97	31.8%
10–18 years	35	11.5%
Weight for age (z-score)	1382	−1.08 (+4.04)
Most recent CD4 count among those alive		
<350 cells/mm^3^	369	27.3%
≥350 cells/mm^3^	983	72.7%
**Treatment**		
Duration of follow-up in months	1511	27.7 (+19.7)
Current ART regimens		
AZT/3TC/NVP	1236	81.5%
Regimens containing d4T	81	5.3%
Other regimen	196	13.0%
**Patients enrolled by facility type**		
Rural	199	13.1%
Peri-urban	201	13.3%
Urban	1116	73.6%
**Quality indicators**		
Screened for tuberculosis at entry into care	1115	81.3%
Adherence counseling documented at last visit	1311	89.7%
Adherence measured at last visit	1296	88.7%
Ever prescribed co-trimoxazole	1482	98.8%
Alive and not lost to follow up with at least one CD4 count in last six months	518	37.0%
Weight documented in chart at patient's last visit	1049	72.2%
High quality indicator score^2^	842	55.5%

1 For patients less than two years of age, severe immunosuppression was defined as an initial CD4 count less than 750 cells/mm^3^ or percentage less than 15%, moderate immunosuppression as an initial CD4 count of between 750 and 1500 cells/mm^3^ or percentage of between 15% and 25%, and no immunosuppression as an initial CD4 count of 1500 cells/mm^3^ or more, or percentage of 25% or more. For patients between two and five years of age, severe immunosuppression was defined as an initial CD4 count less than 500 cells/mm^3^ or percentage less than 15%, moderate immunosuppression as an initial CD4 count of between 500 and 1000 cells/mm^3^ or percentage of between 15% and 25%, and no immunosuppression as an initial CD4 count of 1000 cells/mm^3^ or more, or percentage of 25% or more. For patients between five years of age or older, severe immunosuppression was defined as an initial CD4 count less than 200 cells/mm^3^ or percentage less than 15%, moderate immunosuppression as an initial CD4 count of between 200 and 500 cells/mm^3^ or percentage of between 15% and 25%, and no immunosuppression as an initial CD4 count of 500 cells/mm^3^ or more, or percentage of 25% or more.

2 1 point assigned for each service received (screened for tuberculosis, adherence counseling at last visit, adherence measured by patient/caregiver self-report at last visit, ever prescribed co-trimoxazole, alive and not lost to follow-up with at least one CD4 count in the last six months, and weight documented in chart at patient's last visit, and 0 points assigned if the service was not received, for a total of 6 points. A high score was defined as having the median score or above (>4 points).

The mean duration of follow-up time was 27.7 months (±19.7). Most patients were on an ART regimen comprised of AZT/3TC/NVP (81.5%) at the time of chart review. For those on regimens containing d4T, the mean length of time on the regimen was 36.7 months (±18.5).

### Quality of care

Most patients were screened for tuberculosis at entry into care (81.3%) and had adherence counseling at their last visit (89.7%) (Table 1). Similarly, the majority of patients had their adherence measured at their last visit (88.7%). Almost all patients had been prescribed co-trimoxazole at some point during their enrollment in care (98.8%). Weight for age was documented at the last visit in 72.2% of charts evaluated. However, less than half of patients alive and in care at the time of the chart review had obtained a CD4 count in the six months prior to chart review (37.0%). A higher percentage of patients who were >10 years old were screened for TB compared to those who were <10 years (87.4% vs. 80.3%, p = 0.0148). Over half of patients had a quality score of the median or higher (greater than four points out of six) (55.5%). No significant difference was noted in overall quality score between adolescents (10–18) and younger patients (p = 0.325).

Two quality indicators improved over time. The percentage of patients with a CD4 count in the last six months (p<0.0001) and weight for age documented at the last visit (p = 0.0034) was higher for those with an initial visit in more recent years (2008 to 2011) than for those who enrolled in care prior to 2008.

### Outcomes

Documented mortality within 90 days of ART initiation was 1.9% (Table 2). A total of 4.2% of patients died during the period of follow-up. Mortality was highest for those 24 months old or less at ART initiation (35.9%) (p = NS). Loss to follow-up was 19.0% during the follow-up period, with 3.6% of patients lost within six months of ART initiation and 6.9% lost within the first twelve months. Loss to follow-up was highest for those 25 to 71 months old at ART initiation (35.9%), compared to the other age groups (p = 0.0130). No significant difference was noted in mortality or loss to follow-up between adolescents (age 10–18) and younger patients (p = 0.583 and p = 0.565, respectively).

**Table 2:** Mortality and loss to follow-up by age at ART initiation.

**Mortality**	*n*	*%*	*p value* 1
Within 90 days of ART Initiation	30	1.9%	
During period of follow-up	64	4.2%	
By age at ART initiation			
0–24 months	23	35.9%	NS
25–71 months	20	31.3%	
6–9 years	13	20.3%	
10–18 years	8	12.5%	
**Loss to follow-up**			
Within 6 months of ART Initiation	52	3.6%	
Within 12 months of ART Initiation	100	6.9%	
During period of follow-up	276	19.0%	
By age at ART initiation			
0–24 months	83	30.4%	p = 0.0130
25–71 months	98	35.9%	
6–9 years	48	17.6%	
10–18 years	44	16.1%	

1 Differences between age groups significant at p<0.05.

NS, not significant.

### Bivariate results

**Figure 1:**
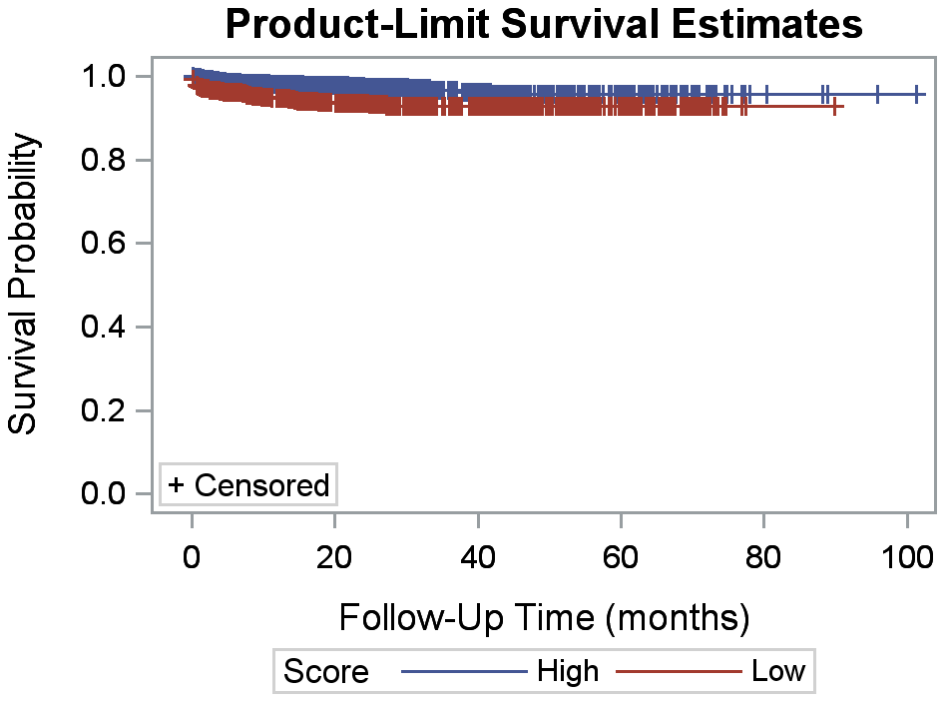
Kaplan-Meier survival by quality indicator score (high vs. low).

**Figure 2:**
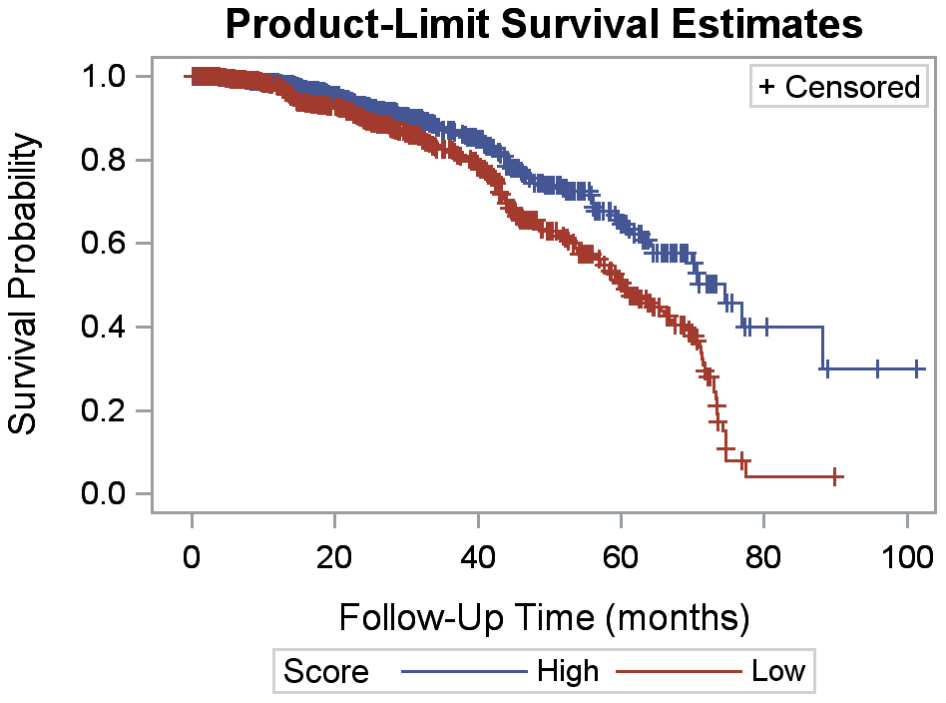
Kaplan-Meier loss to follow-up by quality indicator score (high vs. low).

Patients with a high quality score were more likely to survive over time (p = 0.0011) and less likely to be loss to follow-up (p<0.0001) than patients with a low quality indicator score (Figures 1 and 2). Gender was not statistically significant in bivariate results for mortality or loss to follow-up. A one-point increase in weight for age z-score had a protective effect on mortality (HR: 0.90; 95% CI: 0.85, 0.95) (Table 3). Patients 24 months or younger at ART initiation had a greater likelihood of death (HR: 1.76; 95% CI: 1.06, 2.94) and loss to follow-up (OR: 1.56; 95% CI: 1.16, 2.09), compared to those older than 24 months at ART initiation. Patients with severe immunosuppression were more likely to die (HR: 6.11; 95% CI: 1.89, 19.76) and be lost to follow-up (OR: 1.48; 95% CI: 1.02, 2.14) than patients with no suppression. Overall, a high quality score had a protective effect on mortality (HR: 0.43; 95% CI: 0.26, 0.73) and loss to follow-up (OR: 0.40; 95% CI: 0.31, 0.53) compared to those with a low score.

**Table 3:** Factors associated with mortality and loss to follow-up.

	Mortality			Mortality (n = 1315)		Loss to Follow-up			Loss to Follow-up (n = 1386)	
	*Bivariate*			*Multivariate*		*Bivariate*			*Multivariate*	
	N	HR (95% CI)	p-value	AHR (95% CI)	p-value	N	OR (95% CI)	p-value	AOR (95% CI)	p-value
Male (Ref: Female)	1509	1.50 (0.90, 2.49)	NS	—	—	1450	1.00 (0.77, 1.30)	NS	—	—
Weight for age (z-score)	1379	0.90 (0.85, 0.95)	0.0003	0.92 (0.87, 0.98)	0.0121	1328	0.99 (0.96, 1.03)	NS	—	—
Age at ART initiation ≤24 months (Ref: >24)	1506	1.76 (1.06, 2.94)	0.0297	1.00 (0.51, 1.99)	NS	1447	1.56 (1.16, 2.09)	0.0029	1.36 (0.99, 1.87)	NS
Baseline immunosuppression^1^	1435					1388				
Severe		6.11 (1.89, 19.76)	0.0025	7.21 (1.72, 30.21)	0.0068		1.48 (1.02, 2.14)	0.0374	1.45 (0.99, 2.11)	NS
Moderate		1.95 (0.53, 7.19)	NS	2.88 (0.62, 13.39)	NS		1.14 (0.77, 1.70)	NS	1.13 (0.75, 1.70)	NS
No suppression		*Ref*		*Ref*			*Ref*		*Ref*	
High quality indicator score^2^ (Ref: Low)	1511	0.43 (0.26, 0.73)	0.0015	0.47 (0.26, 0.87)	0.0165	1452	0.40 (0.31, 0.53)	<0.0001	0.42 (0.32, 0.56)	<0.0001

HR, hazard ratio; AHR, adjusted hazard ratio; OR, odds ratio; AOR, adjusted odds ratio; CI, confidence interval; NS, not significant

1 For patients less than two years of age, severe immunosuppression was defined as an initial CD4 count less than 750 cells/mm^3^ or percentage less than 15%, moderate immunosuppression as an initial CD4 count of between 750 and 1500 cells/mm^3^ or percentage of between 15% and 25%, and no immunosuppression as an initial CD4 count of 1500 cells/mm^3^ or more, or percentage of 25% or more. For patients between two and five years of age, severe immunosuppression was defined as an initial CD4 count less than 500 cells/mm^3^ or percentage less than 15%, moderate immunosuppression as an initial CD4 count of between 500 and 1000 cells/mm^3^ or percentage of between 15% and 25%, and no immunosuppression as an initial CD4 count of 1000 cells/mm^3^ or more, or percentage of 25% or more. For patients between five years of age or older, severe immunosuppression was defined as an initial CD4 count less than 200 cells/mm^3^ or percentage less than 15%, moderate immunosuppression as an initial CD4 count of between 200 and 500 cells/mm^3^ or percentage of between 15% and 25%, and no immunosuppression as an initial CD4 count of 500 cells/mm^3^ or more, or percentage of 25% or more.

2 1 point assigned for each service received (screened for tuberculosis, adherence counseling at last visit, adherence measured by patient/caregiver self-report at last visit, ever prescribed co-trimoxazole, alive and not lost to follow-up with at least one CD4 count in the last six months, and weight documented in chart at patient's last visit and 0 points assigned if the service was not received, for a total of 6 points. A high score was defined as having the median score or above (>4 points).

### Multivariate results

In multivariate Cox regression analyses, adjusting for other factors, weight for age z-score (AHR: 0.92; 95% CI: 0.87, 0.98) and high quality score (compared a low score, AHR: 0.47; 95% CI: 0.26, 0.87) had a protective effect on mortality. Patients with severe baseline immunosuppression were more likely to die than those with no immunosuppression (AHR: 7.21; 95% CI: 1.72, 30.21) (Table 3). Adjusting for other factors in multiple logistic regression analysis, patients with a high quality score were less likely to be lost to follow-up (AOR: 0.42; 95% CI: 0.32, 0.56), compared to those with low score.

## Discussion

To our knowledge, this is the first report of a pediatric and adolescent HIV care and treatment quality assessment in Nigeria. Our findings suggest that providing high quality care to children and adolescents living with HIV is associated with lower loss to follow-up and mortality, providing compelling evidence that investing in quality has the potential to retain this age group in care and save lives.

In this study, six process indicators were selected to measure quality. Overall, receipt of services was high. More than 80% of patients were screened for TB at entry into care. However, a higher percentage of adolescents were screened for TB compared to pediatric patients. Though the World Health Organization recommends screening for TB in pediatric patients as described, under-diagnosis and diagnostic delays are common. [38] Intensive training and improved strategies for early TB diagnosis in this age group are needed. Adherence was measured, and adherence counseling was provided to nearly 90% of patients at their last visit. Almost all patients were started on co-trimoxazole at some point since enrollment. Weight for age (z-score) was documented in approximately 70% of charts. The percentage of patients with documented weight for age (z-score) increased in 2008–2011 compared to previous years. Increasing site level experience, improved availability of resources (scales) and training may have contributed to this difference. Low quality scores were largely due to deficits in receipt of CD4 count testing within 6 months of the chart review. The low rates of CD4 count testing may have been due to human resource deficits, dysfunctional equipment, reagent stock-outs, or limited ability to transport samples to a central lab facility, all common challenges identified in a larger assessment of these sites. [File S1]

The quality score devised for this study was significantly correlated with both loss to follow-up and mortality, with a higher score associated with decreased loss to follow-up and increased survival. This finding correlates with limited data from studies in the US demonstrating the association between quality of HIV care and clinical outcomes. [39], [40] In this study, survival may have been lower in patients who did not receive selected process indicators because sub-optimal adherence and treatment failure were missed. Many of the patients who were lost to follow-up may have been too ill to return to the clinic. Higher weight for age z-score had a protective effect on mortality. Numerous studies have also noted this finding. [41]–[44]

Very limited clinical outcomes data are currently available from the Nigerian pediatric and adolescent HIV treatment program. Though the focus of this study was not solely on clinical outcomes, this study provides a reasonable estimate of loss to follow-up and mortality within the Nigerian national program. Early mortality was 1.9% across all age groups, while mortality during the period of follow-up was noted to be 4.2%. Compared to data from similar pediatric and adolescent cohorts in sub-Saharan Africa, mortality was lower in the Nigerian program. [44], [45] Similar to other studies, loss to follow-up was high at 19.0%. [46], [47]

This study has several limitations. Sites were not randomly selected because we wanted to include a geographically diverse sample. Furthermore, we were limited to certain states due to safety concerns. We did not measure all site characteristics that may have impacted the process indicators included in the quality score in this study. This was because our goal was to focus on process, not structure. Furthermore, we knew from a larger assessment that all the sites had similar resource availability. [File S1] Though we provided an estimate of pediatric program staffing, we were unable to account for individual provider characteristics such as years of experience treating pediatric or adolescent patients living with HIV or specialty training which may have impacted performance measures. In addition, as death was only documented in the chart if confirmation was obtained from the patient's family or caregiver or if death occurred in the hospital, it is not possible to know how many patients who were lost to follow up were actually dead. Staff members were trained to contact caregivers when patients were loss to follow-up, but standards regarding the timeliness of this contact were not well defined, and deaths may have been missed. Lastly, accurate measurement of quality is largely dependent upon the accuracy of documentation which is challenging to confirm in a retrospective study.

Pediatric and adolescent access to antiretroviral therapy in Nigeria is expanding. Since 2013, the number of facilities across the country that provide ART to children and adolescents has increased significantly. In order to expedite access to treatment, decentralization or down-referral of pediatric care to primary health clinics has been initiated. Efforts are also underway to standardize services across facilities and to ensure that quality improvement is incorporated into regular in service training. [Federal Ministry of Health of Nigeria, Personal Communication, January 15, 2013] As pediatric and adolescent ART access continues to expand in Nigeria and in similar resource limited settings, ensuring the quality of care that patients receive will be essential to reducing mortality and loss to follow-up. The next logical step toward achieving this goal in the Nigerian treatment program would be to use these data to inform a quality improvement intervention. Quality improvement studies are infrequently published in the medical literature. However, a few studies have demonstrated the efficacy of quality improvement in HIV care and treatment in resource limited settings. [48]–[50] More broadly, quality improvement methods strengthen health systems and help program planners utilize scarce resources more efficiently. [51] Considering the enormous investment that has been made in expanding access to pediatric and adolescent HIV treatment, assessments of quality followed by the development of appropriate interventions are critically important to maximize the benefits of ART.

## Supporting Information

File S1 Greeson D, Ojikutu B, Kolapo U, Higgins Biddle M, Cabral H, et al. (2012) Rapid assessment of pediatric HIV treatment in Nigeria. Arlington, VA: USAID's AIDS Support and Technical Assistance Resources, AIDSTAR-One, Task Order 1. Available at: http://www.aidstar-one.com/focus_areas/treatment/resources/report/pediatric_tx_nigeria. Accessed 2013 Dec 14.
(PDF)
