# Supplementary material for: The Association between Quality of HIV Care, Loss to Follow-Up and Mortality in Pediatric and Adolescent Patients Receiving Antiretroviral Therapy in Nigeria
Source: PLoS One. 2014 Jul 30;9(7):e100039. doi: 10.1371/journal.pone.0100039 (PMC4116117; doi:10.1371/journal.pone.0100039)
Supplement: File S1 — Greeson D, Ojikutu B, Kolapo U, Higgins Biddle M, Cabral H, et al. (2012) Rapid assessment of pediatric HIV treatment in Nigeria. Arlington, VA: USAID's AIDS Support and Technical Assistance Resources, AIDSTAR-One, Task Order 1. Available at: http://www.aidstar-one.com/focus_areas/treatment/resources/report/pediatric_tx_nigeria. Accessed 2013 Dec 14. (PDF) [file pone.0100039.s001.pdf]

# RAPID ASSESSMENT OF PEDIATRIC HIV TREATMENT IN NIGERIA

AIDSTAR-One
AIDS SUPPORT AND TECHNICAL ASSISTANCE RESOURCES

**JANUARY 2013**

This publication was made possible through the support of the U.S. President's Emergency Plan for AIDS Relief (PEPFAR) through the U.S. Agency for International Development under contract number GHH-I-00-07-00059-00, AIDS Support and Technical Assistance Resources (AIDSTAR-One) Project, Sector I, Task Order 1.

# RAPID ASSESSMENT OF PEDIATRIC HIV TREATMENT IN NIGERIA

The authors' views expressed in this publication do not necessarily reflect the views of the U.S. Agency for International Development or the United States Government.

**AIDS Support and Technical Assistance Resources Project**
AIDS Support and Technical Assistance Resources, Sector I, Task Order 1 (AIDSTAR-One) is funded by the U.S. President's Emergency Plan for AIDS Relief (PEPFAR) through the U.S. Agency for International Development (USAID) under contract no. GHH-I-00–07–00059–00, funded January 31, 2008. AIDSTAR-One is implemented by John Snow, Inc., in collaboration with Broad Reach Healthcare, Encompass, LLC, International Center for Research on Women, MAP International, Mothers 2 Mothers, Social and Scientific Systems, Inc., University of Alabama at Birmingham, the White Ribbon Alliance for Safe Motherhood, and World Education. The project provides technical assistance services to the Office of HIV/AIDS and USG country teams in knowledge management, technical leadership, program sustainability, strategic planning, and program implementation support.

**Recommended Citation**
Dana Greeson, Bisola Ojikutu, Usman Kolapo, Molly Higgins-Biddle, Howard Cabral, and Ellen Cooper. 2013. *Rapid Assessment of Pediatric HIV Treatment in Nigeria*. Arlington, VA: USAID's AIDS Support and Technical Assistance Resources, AIDSTAR-One, Task Order 1.

**Abstract**
Early access to antiretroviral therapy (ART) is particularly important for HIV-infected children. In Nigeria, a disparity exists between pediatric and adult ART coverage, with pediatric and adult coverage rates at 7 percent and 26 percent, respectively (WHO 2011a). To better understand barriers to pediatric treatment scale-up, AIDSTAR-One conducted an assessment of 23 treatment sites throughout Nigeria. Part I of the assessment explores site-level barriers to providing high quality pediatric HIV care and treatment services. Part II provides an overview of clinical outcomes of the children and adolescents treated at these sites and describes the impact of health systems characteristics explored in Part I on outcomes and quality of care.

**Part I** – AIDSTAR-One developed a comprehensive Site Assessment Tool and Walk-through Checklist for administration at the selected sites. Analysis of the data collected highlighted a number of significant barriers to the provision of quality pediatric HIV treatment. These included human resources constraints, lack of caregiver involvement, lack of disclosure, antiretroviral adherence issues, limited adolescent-specific care, difficulties with data management, and inadequate resources onsite. Recommendations for mitigating these barriers and improving the quality of pediatric HIV care and treatment are offered in this document.

**Part II** – AIDSTAR-One conducted a retrospective chart review including 1,516 pediatric and adolescent patients enrolled in care from 2002 to 2011 at the 23 sites surveyed. Data collected included age, weight for age (z-score), ART regimen, follow-up, and mortality. The project performed descriptive analyses and examined associations with survival and loss to follow-up using Cox proportional hazards and logistic regression. During the period of follow-up (mean 24.4 months), 4.2 percent of patients died. Loss to follow-up was 19.1 percent. Overall, treatment outcomes in the Nigerian program were encouraging. Few health systems challenges noted in Part I impacted outcomes of treatment. Interventions to ensure earlier access to ART and decrease loss to follow-up are warranted.

**Acknowledgments**
The authors would like to thank the following agencies for providing input and guidance in development of this assessment: the U.S. Agency for International Development (USAID)/Washington, USAID/Nigeria, the Nigerian Federal Ministry of Health, Indepth Precision Consult Limited, AIDSRelief, AIDS Prevention Initiative in Nigeria/Harvard, FHI/Global HIV/AIDS Initiative Nigeria, Institute of Human Virology Nigeria, International Center for AIDS Care and Treatment Programs, Management Sciences for Health Prevention and Organizational Systems AIDS Care and Treatment, and Christian Health Association of Nigeria. The authors would also like to thank Benjamin R. Phelps (USAID) and Anouk Amzel (USAID) of the U.S. President's Emergency Plan for AIDS Relief Treatment Technical Working Group, Emeka Okechukwu (USAID/Nigeria), Azeez Aderemi (Federal Ministry of Health), and all of the respondents who participated in the assessment.

**AIDSTAR-One**
John Snow, Inc.
1616 Fort Myer Drive, 16th Floor
Arlington, VA 22209 USA
Phone: 703-528-7474
Fax: 703-528-7480
E-mail: info@aidstar-one.com
Internet: aidstar-one.com

# CONTENTS

**Acronyms** ..... **vii**

**Executive Summary** ..... **ix**

Part I ..... ix

Part II ..... xi

**Introduction** ..... **1**

Pediatric HIV and Treatment ..... 1

Pediatric HIV and Treatment in Nigeria ..... 2

Nigeria's 2010 Pediatric ART Guidelines ..... 2

**Part I: Methodology** ..... **5**

Assessment Tools Development ..... 5

Training and Pilot ..... 5

Role of Federal Ministry of Health and State Ministry of Health ..... 6

Onsite Rapid Assessments ..... 6

Limitations ..... 6

**Part I: Results** ..... **7**

Human Resources ..... 7

Staff Training ..... 7

Guidelines and Protocols ..... 8

HIV Care (Pre-ART) ..... 8

ART Eligibility and Initiation ..... 9

ART ..... 9

Monitoring and Evaluation ..... 9

Service Delivery Models ..... 10

Service Integration ..... 11

Adolescent Services ..... 12

HIV-exposed Infants ..... 13

Adherence ..... 13

Defaulters ..... 14

TB/HIV Co-infection ..... 14

Supportive Services ..... 15

Nutrition ..... 16

Community Engagement ..... 17

Pharmacy ..... 17

Laboratory ..... 18

Early Infant Diagnosis 18
Walk-through Checklist 20

**Part I: Overview and Recommendations 23**

Human Resources 23
Caregivers 23
Disclosure 23
Adherence 24
Adolescents 24
Data Management 24
Site Resources 25

**Part II: Methodology, Results, and Conclusion 27**

Methodology 27
Results 29
Results Summary 33
Conclusion 34

**References 35**

**Appendix A 37**

**Appendix B 39**

**Appendix C 41**

**Appendix D 43**

**Figures**

Figure 1. Map of Nigerian States. 4
Figure 2. Services Offering Pediatric HIV Testing & Counseling 10
Figure 3. Entry Point for Exposed Infants and EID Integration 19

**Tables**

Table 1. Topics Covered by Site Assessment Tool 5
Table 2. Training Topics Covered at Sites 7
Table 3. Service Integration 11
Table 4. Services Tailored to Adolescents by Site 12
Table 5. Psychosocial Support Services Offered by Site 15
Table 6. Growth Monitoring Methods Used by Site 16
Table 7. Decision Aids Available by Site 20
Table 8. Supplies/Equipment Available at Sites 21
Table 9. Characteristics of Sampled Pediatric and Adolescent Patients in Nigerian Treatment Program 30
Table 10. Quality Data 31
Table 11. Bivariate Analysis of Immunosuppression and Quality Indicators 31
Table 12. Outcomes from the Pediatric HIV Treatment Program 32
Table 13. Factors Associated with Death and Loss to Follow-Up 33

# ACRONYMS

| | |
|---|---|
| 3TC | lamivudine |
| AFASS | acceptable, feasible, affordable, sustainable, and safe |
| ALHIV | adolescents living with HIV |
| ANC | antenatal care |
| ART | antiretroviral therapy |
| ARV | antiretroviral |
| ASW | adherence support worker |
| AZT | zidovudine |
| CBO | community-based organization |
| CD4 | cluster of differentiation 4 |
| CDC | U.S. Centers for Disease Control and Prevention |
| CTX | cotrimoxazole |
| d4T | stavudine |
| DBS | dried blood spot |
| DHMT | District Health Management Team |
| DMO | District Medical Officer |
| EFV | efavirenz |
| EID | early infant diagnosis |
| EPI | Expanded Programme in Immunization |
| FDC | fixed dose combination |
| FMoH | Federal Ministry of Health |
| GOPD | general outpatient department |
| HR | human resources |
| IMCI | integrated management of childhood illness |
| INH | isoniazid |
| IP | implementing partner |
| LFT | liver function test |
| LPV/r | lopinavir/ritonavir |
| MDG | Millennium Development Goal |

| | |
|---|---|
| NHREC | National Health Research Ethics Committee of Nigeria |
| NNRTI | non-nucleoside reverse transcriptase inhibitor |
| NRTI | nucleoside reverse transcriptase inhibitor |
| NVP | nevirapine |
| OVC | orphans and vulnerable children |
| PCR | polymerase chain reaction |
| PLHIV | people living with HIV |
| PMO | Provincial Medical Officer |
| PMTCT | prevention of mother-to-child transmission |
| SAPC | State HIV/AIDS Programme Coordinator |
| SMOH | State Ministries of Health |
| SMS | short message service |
| TAT | turnaround time |
| TB | tuberculosis |
| TWG | technical working group |
| WHO | World Health Organization |
| UNAIDS | Joint United Nations Programme on HIV/AIDS |
| UNICEF | United Nations Children's Fund |

# EXECUTIVE SUMMARY

Antiretroviral therapy (ART) has averted an estimated 2.5 million deaths in resource-limited settings since 1995 (WHO 2011a). Early access to antiretrovirals (ARVs) is particularly important for HIV-infected children whose naturally underdeveloped immune systems increase their vulnerability to opportunistic infections as well as common, but severe, childhood illnesses. Nigeria faces a generalized HIV epidemic with a prevalence of 3.6 percent in 2009 (WHO 2011a). The prevalence rate and population density in Nigeria combine to make it home to the second largest number of people living with HIV in the world after South Africa (WHO 2011a). In 2009, approximately 3,300,000 people in Nigeria were living with HIV, including 360,000 children (UNAIDS 2010). As is the case globally, a disparity exists between pediatric and adult ART coverage in Nigeria, with pediatric and adult ART coverage rates at 7 percent and 26 six percent, respectively (WHO 2011a).

Following the World Health Organization's (WHO) 2010 revision of "Antiretroviral Therapy for HIV Infection in Infants and Children: Towards Universal Access," the Nigerian Federal Ministry of Health (FMoH) released a national adaptation, "National Guidelines on Paediatric HIV and AIDS Treatment and Care." The revised national guidelines call for improved pediatric HIV treatment through measures such as initiating all HIV-positive children younger than two years of age on treatment, irrespective of clinical or immunological status (Federal Ministry of Health [FMoH] 2010).

In 2011, AIDSTAR-One conducted a rapid assessment of pediatric HIV treatment scale-up in Nigeria to better understand the barriers to providing and expanding high quality pediatric HIV care and treatment services. The assessment was funded by the PEPFAR's Pediatric Treatment Technical Working Group (TWG). AIDSTAR-One worked in collaboration with the Nigerian FMOH and USAID/Nigeria. AIDSTAR-One hired Indepth Precision Consult Limited as a local consulting organization tasked with finding local data capturers and overseeing day-to-day assessment logistics.

The assessment was divided into two components. In Part I, AIDSTAR-One developed and administered a comprehensive Site Assessment Tool and Walk-through Checklist at 23 sites in order to identify barriers to the delivery of high quality pediatric and adolescent HIV/AIDS care and treatment services in Nigeria. In Part II of the assessment, AIDSTAR-One conducted a retrospective chart review of 1,516 pediatric and adolescent patients enrolled in care from 2002 to 2011 at the 23 sites surveyed in order to determine outcomes of care.

## PART I

Specific Objectives:

1. Identify barriers and facilitators to the delivery of high quality pediatric and adolescent HIV/AIDS care and treatment services in Nigeria.

2. Assess resources available and necessary to implement and expand pediatric treatment services.

The Federal Ministry of Health selected 23 pediatric ART sites across 10 states for the assessment (see Appendix A). The study protocol and assessment tools were submitted to the National Health Research Ethics Committee of Nigeria (NHREC) and approved on April 9, 2011. AIDSTAR-One performed a desk review and developed a Site Assessment Form and Walk-through Checklist for administration at the 23 sites. These tools covered the following domains: human resources, treatment guidelines, ART eligibility and initiation, adolescent services, adherence, nutrition, community engagement, and pharmacy and laboratory services.

AIDSTAR-One facilitated a three-day training session with six data collectors, which included a pilot at three sites in Abuja. In concordance with FMOH protocol, teams visited the State HIV/AIDS Program Coordinators (SAPC) in each location to explain the goals of the assessment and seek permission for site visits.

Analysis of the data collected in the AIDSTAR-One tools and by implementing partners highlighted the most prominent barriers to providing quality pediatric HIV treatment. These included:

- Persistent human resources constraints, most notably the standard reassignment of trained personnel
- The inability of some caregivers to meet patients' needs
- Lack of pediatric and adolescent disclosure
- Patient and caregiver difficulty adhering to ART
- Limited availability of targeted care, treatment, and support programs for adolescents
- Data management challenges
- Inadequate resources (equipment, tools, etc.) at the facility

A number of strategies to overcome the barriers noted have been proposed:

***Human resources*** – Limiting the number of trained personnel who were transferred to other sites would go a long way in mitigating HR challenges, allowing facilities to better serve their patients.

***Caregiver support*** – Support systems must be improved for caregivers of pediatric and adolescents living with HIV. Similar to the mentor mother model initiated by mothers2mothers, AIDSTAR-One suggests a mentor caregiver initiative whereby attentive (nonparental) caregivers mentor those who are struggling, through psychosocial support and advice.

***Disclosure*** – A number of resources are available online. One helpful resource for providers is included in the International Center for AIDS Care and Treatment Programs' "Adolescent HIV Care and Treatment: A Training Curriculum for Multidisciplinary Health Care Teams." Module 7 is on "Providing Disclosure Counseling and Support." Available at: http://www.columbia-icap.org/resources/supporttools/index.html.

***Adherence*** – Children at various ages can be provided with pill calendars and can be encouraged to personalize them or make their own. This is an inexpensive way for children and adolescents living with HIV to learn medication management.

***Adolescents*** – With the increasing population of adolescents living with HIV, more clinics should consider targeting services to this group. AIDSTAR-One has created an Adolescent Toolkit for providers of ALHIV, which will be posted on the AIDSTAR-One website in the coming months.

The Toolkit address issues such as disclosure and dating and provides guidance on psychosocial support.

*Data Management* – Implementing partners and/or individual sites that are successfully managing patient data electronically should be asked to share best practices in electronic medical records (EMR) training and implementation with the FMOH for use with other implementing partners (IPs)/sites that are ready to make the transition to EMR. The successful expansion of EMRs across more sites would also serve to improve data collected at the national level.

# PART II

Part II of this study describes pediatric and adolescent outcomes in Nigeria and explores the impact of site characteristics on loss to follow-up and mortality. A retrospective chart review was conducted including a systematically sampled cohort of patients who initiated ART from 2002 to 2011. The sample included 1,516 patients from the 23 assessment sites. Data collected included age, weight for age (z-score), ART regimen, visit dates and mortality, and several quality indicators. Overall, mean age at initiation was 64 months (standard deviation [SD] 51.7). Severe immunosuppression (see Appendix A) was noted in 45 percent (653) at enrollment. Mean z-score was –1.1 (SD 4.0). Median duration of follow-up was 24.4 months. Mean time from ART eligibility to ART initiation was 26.2 weeks (SD 43.0). The most common initial regimen was AZT/3TC/NVP (81.5 percent). Mortality within 90 days of ART initiation was 1.9 percent. A total of 4.2 percent died during the period of follow-up (mean 24.4 months). Loss to follow-up was 19.1 percent.

In bivariate results, weight for age z-score, moderate to severe immunosuppression, and receiving care at a rural treatment site were significantly associated with mortality. In multivariate results, adjusting for other factors, moderate to severe immunosuppression and rural treatment site remained as predictors of mortality. Moderate to severe immunosuppression, age younger than two years, and a delay to ART initiation of greater than 12 weeks were significantly associated with loss to follow-up. In multivariate results, adjusting for other factors, both age younger than two years and a delay to ART initiation greater than 12 weeks remained significant.

Overall, treatment outcomes in the Nigerian program were encouraging. Few health system challenges noted in Part I impacted outcomes of treatment. Interventions to ensure earlier access to ART and decrease loss to follow-up are warranted.

# INTRODUCTION

## PEDIATRIC HIV AND TREATMENT

Antiretroviral therapy (ART) has averted an estimated 2.5 million deaths in resource-limited settings since 1995 (WHO 2011a). Combination ART has revolutionized the HIV epidemic by increasing the lifespan of people living with HIV (PLHIV), enhancing quality of life, and decreasing transmission rates by reducing viral load. HIV treatment is no longer universally synonymous with daily pill burden and harsh side effects. Fixed dose combinations (FDCs)—two or more active antiretrovirals (ARVs) combined in a single fixed dose—have improved ART adherence through decreased pill burden, more manageable dosing schedules, and fewer side effects and interactions.

Several global initiatives have set goals to expand ART coverage among those populations most vulnerable to HIV. In 2003, the World Health Organization (WHO) and the Joint United Nations Program on HIV/AIDS' (UNAIDS) "3 by 5" initiative aimed to reach three million people worldwide with ART by the end of 2005. When the initiative began, only 400,000 people were on ARVs globally. The goal of reaching three million people was eventually achieved in 2007. By the end of 2010, 6.65 million people were on ART (WHO 2011a). In 2000, the Millennium Development Goals (MDGs) were developed. Goal 6B aimed to achieve universal access to HIV treatment for persons in need by 2010. The impetus for the inclusion of this goal was due to infections continuing to surpass treatment scale-up, and evidence indicating that treatment expansion for HIV-positive women of childbearing years could also protect their babies (UN MDGs 2011).

Between 2007 and 2010, the global number of clinical care facilities providing ART tripled (from 7,700 to 22,400), and ART coverage increased 16-fold from 2003 to 2010 (WHO 2011a). Despite this commendable scale-up effort, less than half of the people eligible for treatment were accessing ARVs by the end of 2010. In 2009, the number of children (under 15 years of age) living with HIV reached 2.5 million (UNAIDS 2010). Although global adult ART coverage is 51 percent, ART coverage for children is at only 23 percent (456,000 children). The disparity between pediatric and adult ART coverage has been associated with complicated diagnostics and weight-based dosing, a rigorous cascade of care, unavailability of pediatric ART formulations, and the later implementation of pediatric HIV programs and drug regimens in health facilities. Concerning attrition rates along the entire pediatric cascade of care emphasize the importance of additional community supports and linkages within the health system (WHO 2011a).

Early access to ARVs is particularly important for HIV-infected infants whose naturally underdeveloped immune systems increase their vulnerability to opportunistic infections as well as common, but severe, childhood illnesses. Without early diagnosis and treatment, approximately one-third of HIV-infected infants will die before their first birthday and almost half before their second birthday. Appropriate diagnostics and treatment coupled with strong adherence make it possible for HIV-infected infants to reach adolescence and adulthood (WHO 2011a).

# PEDIATRIC HIV AND TREATMENT IN NIGERIA

Nigeria faces a generalized HIV epidemic with a prevalence of 3.6 percent in 2009 (WHO 2011a). The prevalence rate and population density in Nigeria combine to make it home to the second largest number of people living with HIV in the world after South Africa (WHO 2011a). In 2009, approximately 3,300,000 people in Nigeria were living with HIV, including 360,000 children (UNAIDS 2010). ART coverage in Nigeria was reported at 26 percent, with 359,181 people receiving ART. This coverage rate increased 19 percent from 2009. In 2010, people on ART in Nigeria accounted for 5 percent of all people on ART in low- and middle-income countries (WHO 2011a). Similar to the global situation, there is a disparity between pediatric and adult ART coverage in Nigeria with coverage rates at 7 percent and 26 percent, respectively (WHO 2011a).

# NIGERIA'S 2010 PEDIATRIC ART GUIDELINES

Following the World Health Organization's (WHO) 2010 revision of "Antiretroviral Therapy for HIV Infection in Infants and Children: Towards Universal Access," the Nigerian Federal Ministry of Health (FMoH) released a national adaptation to previous guidelines, "National Guidelines on Paediatric HIV and AIDS Treatment and Care." The updated guidelines are intended to:

1. Provide standardized management protocols based on current evidence for children infected with or exposed to HIV infection.

2. Provide guidance for monitoring and evaluation of comprehensive pediatric HIV and AIDS services.

3. Serve as a reference document to advisory boards, program managers, and other policymakers involved in pediatric HIV/AIDS programming.

In accordance with the updated guidelines, health care providers are instructed to initiate all HIV-positive children younger than 24 months of age on ART. Children ≥ 24 months with a positive rapid test are initiated on ART based on immunologic criteria (WHO clinical stage is 3 or 4 irrespective of CD4 count or CD4%; CD4 count is ≤ 750 cells/mm3 or ≤ 25% in children aged 24–59 months with WHO clinical stage 1 or 2; and CD4 count is ≤350 cells/mm3 or ≤ 25% in children ≥ 5 years with WHO clinical stage 1 or 2). Previous guidelines (2007) recommended using immunologic criteria to determine initiation for all children irrespective of age.

The recommended first-line regimen in Nigeria for children younger than three years of age is a non-nucleotide reverse transcriptase inhibitor (NNRTI)–based regimen comprised of nevirapine (NVP) and zidovudine (AZT) + lamivudine (3TC). Children older than three years of age are started on either efavirenz (EFV) or NVP and AZT + 3TC. If exposure to NVP for prevention of mother-to-child transmission (PMTCT) is documented, then the guidelines recommend starting a protease inhibitor–based regimen including lopinavir/ritonavir. During the period of this study, children may have initiated alternative regimens based upon previous guidelines or the presence of Mycobacterium tuberculosis (MTB) co-infection.

## OVERVIEW

In October 2011, AIDSTAR-One conducted a rapid assessment of pediatric HIV treatment scale-up in Nigeria to better understand the barriers to providing and/or expanding high quality pediatric HIV care and treatment services. The assessment was funded by PEPFAR's Pediatric Treatment Technical Working Group (TWG). AIDSTAR-One worked in collaboration with the Nigerian FMoH and USAID/Nigeria, and hired Indepth Precision Consult Limited as a local consulting organization tasked with hiring local data capturers and overseeing day-to-day assessment logistics.

The assessment was divided into two components. In Part I, AIDSTAR-One developed and administered a comprehensive Site Assessment Tool and Walk-through Checklist at 23 sites in October 2011. The goal of Part I of this assessment was to identify barriers to the delivery of high quality pediatric and adolescent HIV/AIDS care and treatment services in Nigeria. In Part II of the assessment, AIDSTAR-One conducted a retrospective chart review of 1,516 pediatric and adolescent patients enrolled in care from 2002 to 2011 at the 23 sites surveyed in order to determine outcomes of care and explore the impact of site characteristics on loss to follow-up and mortality (please see Part II for detailed chart review methodology).

## ETHICAL REVIEW

AIDSTAR-One's rapid assessment of pediatric treatment scale-up was conducted in October 2011. The study protocol and assessment tools were submitted to the National Health Research Ethics Committee of Nigeria (NHREC) and approved on April 9, 2011. Informed consent was obtained prior to site survey administration for Part I. Individual signed, informed consent was waived for Part II (the retrospective chart review). Dr. Wapada Balami, the National AIDS Coordinator, notified the State AIDS Program Coordinators (SAPCs) of this activity in advance of the start of the assessment. The SAPCs, in turn, sent letters to the Medical Superintendents of the selected assessment sites to grant permission for the assessments to commence.

## SITE SELECTION

The FMoH used purposive sampling to select 23 pediatric ART sites across 10 states for the assessment (see Appendix C). The criteria guiding site selection included geographic location (Figure 1), setting (i.e., urban, peri-urban, or rural), and site type (i.e., primary, secondary, and tertiary hospitals). Selected sites also varied in terms of which implementing partner (IP) they received support from. IPs included AIDSRelief, AIDS Prevention Initiative in Nigeria/Harvard, FHI/Global HIV/AIDS Initiative Nigeria, Institute of Human Virology Nigeria, International Center for AIDS Care and Treatment Programs, Management Sciences for Health Prevention and Organizational Systems AIDS Care and Treatment, or Christian Health Association of Nigeria.

**Figure 1. Map of Nigerian States**

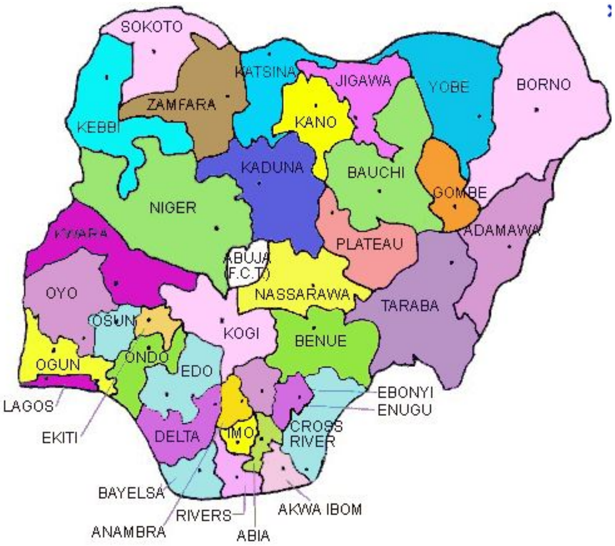


Source: www.nigerianbern.org/states_in_nigeria.htm

# PART I: METHODOLOGY

Part I of this report will detail site assessment methodology, findings, and programmatic recommendations.

## ASSESSMENT TOOLS DEVELOPMENT

AIDSTAR-One developed two tools for this assessment: the Site Assessment Tool and the Walk-through Checklist. Prior to developing the assessment tools, AIDSTAR-One conducted a comprehensive review of key program documents, including national guideline; relevant treatment protocols; and national and site program data, reports, and abstracts. National IPs and site staff were interviewed to provide contextual data and "gray literature," including any additional reports and internal documents as available. The medical literature was reviewed for pertinent published documents to guide the tool development.

### SITE ASSESSMENT TOOL

The Site Assessment Tool contains both quantitative and qualitative questions covering 10 domains (Table 1). The tool was designed to be administered to various health care providers of HIV-infected children (i.e., medical and clinical officers, pediatric HIV nurses, laboratory technologists or technicians, and pharmacists or pharmacy technicians).

**Table 1. Topics Covered by Site Assessment Tool**

| | |
|---|---|
| Human resources | Nutrition |
| Treatment guidelines | Community engagement |
| ART eligibility and initiation | Pharmacy services |
| Adolescent services | Laboratory services |
| Adherence | Early infant diagnosis |

### WALK-THROUGH CHECKLIST

The Walk-through Checklist was designed for data capturers to conduct an independent assessment of available resources and tools. The goal was to determine the resources and space available to pediatric HIV providers and patients. Topics include basic resources (i.e., water, electricity, and telephone), decision aids, pediatric equipment, and "pediatric friendly" physical space.

## TRAINING AND PILOT

Prior to data collection, AIDSTAR-One facilitated a three-day training session with six data collectors in Abuja, Nigeria. Three of the six data collectors were nurses who worked in HIV care, while the remaining three had experience working on HIV programs and research studies. The training curriculum reviewed pediatric HIV and treatment in the global and Nigerian contexts and covered assessment objectives, methodology, and logistics. Assessment tools were reviewed in detail with an in-class role-play and a pre- and post-training assessment test.

On the second training day, the group divided into three teams (each with a nurse) and visited three separate pediatric ART facilities in Abuja to pilot the tools. The following day, teams regrouped to discuss lessons learned, seek clarification of objectives and methodology, provide feedback, and give input into the revision of the tools. The groups also reviewed the Access database and input the data collected from the pilot facilities.

## ROLE OF FEDERAL MINISTRY OF HEALTH AND STATE MINISTRY OF HEALTH

Prior to the training date, the FMoH wrote to the various State Ministries of Health (SMoH) about the assessment that would take place in their domain. The SMoH in turn informed the specific health facilities of the assessment and expected visit date. On arrival at each state, the team visited the SAPCs who are the state representatives on this project. Teams presented a copy of the FMoH letter and briefed the SAPCs on the assessment and asked permission to visit the facilities on the scheduled dates. The teams also visited the contact person at each IP that supported an assessment site(s). The various facilities, SMoH, and the ART focal persons mentioned their desire to receive the assessment results in order to inform pediatric HIV programs at their sites.

## ONSITE RAPID ASSESSMENTS

Upon arrival at each facility, the teams presented the IP representative and the ART focal person with copies of the FMoH and SMoH approval letters and reviewed the assessment protocol. The point of contact introduced teams to the appropriate respondents for the site assessment form. Teams were flexible with their schedules and mindful of respondents' workloads so as to minimize interruptions to patient care. Following introductions and explanations of the aforementioned forms to the respondent, an AIDSTAR-One team member read the informed consent and the respondent provided verbal consent. While administering the Site Assessment Form, one team member read the questions aloud, while another wrote the answers on a separate hard copy of the form. The third team member conducted the Walk-through Checklist while the interviews occurred. Prior to leaving the sites, team leaders reviewed Site Assessment Forms and Walk-through Checklists to ensure clarity and completion. After the site visits, team leaders entered the data into a Microsoft Access Database (Microsoft, Inc., Redmond, WA).

## LIMITATIONS

This assessment has several limitations. The sample size utilized is small and was limited to 10 of the 36 Nigerian states. Data was collected from health care providers who were readily available onsite. Data collectors were instructed to be cognizant of providers' workloads and to be flexible in scheduling meeting times. In some cases, the optimal person to speak with at the facility was unavailable. Since data collectors had a limited number of days in each location, they captured data from alternate individuals. Although these limitations exist, this report provides some insight into pediatric treatment challenges in Nigeria. A larger study would be necessary to ensure generalizability.

# PART I: RESULTS

## HUMAN RESOURCES

Human resource constraints are well recognized throughout health care systems in resource limited settings, and Nigeria is no exception. Eighteen of the 23 sites reported that retention of staff is a significant problem within their pediatric HIV program. The most commonly reported reason for staff departure was standard reassignment (n = 15) followed by funding constraints (n = 6). Several respondents stated that staff had reached out to the hospital management board, the commissioner of health, and/or the state government to discuss the challenges created by frequent staff reassignment. According to respondents, the State Hospital Management Board has prevented the transfer of some trained staff members in response to facility requests. However, retention of sufficient staff to meet patient needs continues to be an ongoing concern.

## STAFF TRAINING

In light of high staff turnaround and revised pediatric treatment protocols, trainings are an important means of keeping staff informed of best practices in providing high quality treatment to pediatric HIV patients. Sites were asked whether specific topics had been included in recent trainings (within the last year) attended by pediatric HIV staff members. Responses are presented in Table 2.

**Table 2. Training Topics Covered at Sites**

| Training Topic | Number of sites reporting inclusion of topic in recent trainings (%) |
|---|---|
| Growth and nutrition assessment | 17 (74%) |
| Physical examination | 17 (74%) |
| Assessing developmental progress | 14 (61%) |
| Ongoing ART eligibility assessments for HIV-infected children not yet on ART | 17 (74%) |

Respondents were asked to identify gaps within the pediatric training program at their site. Several respondents requested trainings in pediatric developmental assessments. Most sites reported that they desire more training in pediatric HIV care in general. Other respondents desired instruction on providing youth-friendly services to adolescents.

Nineteen site respondents (83%) reported that clinical mentoring is offered to pediatric HIV staff. Thirteen of these sites (68%) rated the clinical mentoring as very effective, while the remaining six (26%) rated it moderately effective.

# GUIDELINES AND PROTOCOLS

As noted, in 2010, Nigeria revised their national pediatric treatment guidelines. The new guidelines were printed in October 2010 and were issued to implementing partners at that time. Eighteen sites (78%) reported following Nigeria's 2010 guideline recommendations in their entirety, whereas three (13%) reported following some of the new recommendations. The remaining two sites had not received copies of the updated guidelines and noted that they would implement them once received. Sites reporting partial implementation of the updated guidelines had not yet been able to initiate all HIV-positive infants and children younger than two years on ART.

Sites reported a variety of training methods used to implement the 2010 guidelines. Implementing partners typically provided a comprehensive training for their sites. In some cases, the senior clinicians (e.g., Resident Doctors and Medical Officers) at a site attended a training prior to engaging additional staff in a step-down training. Some sites disseminated copies of the guidelines to each relevant health care worker with key issues highlighted.

Challenges to guideline implementation included stock outs of first-line and second-line ART and reagents making it impossible to provide the optimal services outlined in the guidelines. Staffing issues such as strikes, inadequate training, frequent staff transfers, and a shortage of doctors also make the guidelines challenging to implement. As noted, two sites simply had no copies of the updated guidelines.

For sites that were able to implement all or some of the guidelines, respondents reported that patient quality of care improved after guideline implementation. The updated guidance on breastfeeding—that is, exclusive breastfeeding for the first 6 months followed by continued breastfeeding with complimentary feeding from 6 months to 12 months—reduced malnutrition among infants, according to respondents. Some respondents mentioned that the updated PMTCT guidelines give HIV-infected pregnant and breastfeeding women increased access to ART, resulting in healthier mothers who are better able to provide for their young children. Sites also mentioned that they were encouraged by the recommended introduction of an adolescent treatment and support program.

# HIV CARE (PRE-ART)

Pre-ART care spans from HIV diagnosis to the initiation of ART. During this period, infants, children, and adolescents are at high risk of death and loss to follow-up. Therefore, it is critical that treatment sites provide supportive services and adjunctive therapy to promote retention in care. All 23 sites reported providing cotrimoxazole (CTX) prophylaxis to pre-ART patients. Ninety-nine percent of pediatric ART patients sampled in the chart review had been prescribed CTX at some point during their care (see Part II). Over 80 percent of sites also provided clinical services, nutrition counseling, food supplements, psychosocial support, and counseling to these patients. Three sites (13%) sites added that they make special provisions for orphans and vulnerable children (OVCs), including providing clothing and/or financial and nutritional support.

Respondents noted a number of challenges to providing care to HIV-positive children who are not yet eligible for ART. The most commonly mentioned challenge was loss to follow-up and missed appointments with providers speculating that loss to follow-up is higher among pre-ART patients because they are asked to return for clinical visits, but not to pick up ART. Respondents suggested that caregivers may need more education regarding the importance of follow-up during the pre-ART period. Without the incentive of receiving life-prolonging drugs at facility visits, caregivers may feel

that barriers such as long distances, transportation costs, and missing work outweigh the perceived benefits of the visit. Several providers mentioned that there is sometimes pressure from caregivers who ask that their children be initiated on ART despite ineligibility. Sites also noted that laboratory delays and caregivers' inabilities to pay for certain laboratory tests have delayed ART initiation and, in some cases, compromised patient care.

# ART ELIGIBILITY AND INITIATION

Nineteen sites reported initiating all children younger than two years of age on ART, irrespective of immunological status (83%). Most sites reported initiation criteria in line with the 2010 guidelines. Nineteen sites also reported initiating children on ART based on clinical staging when CD4 counts/percentages were not available.

Twelve sites reported an average of less than two weeks from eligibility for ART to initiation. Seven sites reported an average of between two and four weeks, three took more than a month, and one was unsure.

# ART

Reported barriers to the provision of pediatric ART were a shortage of trained staff, drug stock outs, lack of space, failure of caregivers to take referred patients to specialist centers (sometimes due to transport costs), inability to perform viral loads, and malnutrition—which respondents felt was especially prevalent among orphans. Respondents were most encouraged by the general improvements in the health of their patients as advances in pediatric ART continue to be implemented at the site level.

Nine sites reported the ability to dispense pediatric medications to caregivers without the child present whereas 14 sites reported that the pediatric patient had to be present at each drug pick up. In cases of suspected or confirmed treatment failure, nine sites reported referring patients to specialist centers and the same number of assessment sites reported being specialist centers themselves. Five sites reported that a treatment expert visited on a regular basis.

# MONITORING AND EVALUATION

In regard to monitoring and evaluation, most sites reported using some combination of EMR, patient paper files, patient-held cards, and/or registers. Half of the sites (n = 12) use EMRs. All of these sites also recorded patient information in registers and 11 also used patient paper files. Nine of the sites using EMRs also utilized patient-held cards which include information on demographics, enrollment, ART initiation—including eligibility criteria (see Appendix B) met and initial regimen, substitutions or changes in regimen, and any discontinuations or interruptions in treatment. Overall, 21 sites used patient paper file and/or registers. Seventeen sites utilized patient-held cards.

Most sites (n = 22) reporting have a functioning Quality Improvement Team. These teams were located either external to the site or were based at the site. Twenty sites (87%) reported that pediatric HIV program staff had been trained on quality improvement.

# SERVICE DELIVERY MODELS

Sites reported using a variety of service delivery models. Twelve sites reported seeing pediatric and adult ART patients during the same time in the same place. Six sites reported using the same space to see pediatric and adult ART patients but at different times. Three sites contained a separate area where the pediatric clinic was held.

Mobile clinics provide HIV patients with increased accessibility to services. Thirty-nine percent of sites reported providing some type of pediatric HIV service through mobile clinics. Of these nine sites, HIV rapid testing and counseling was the most common service provided (n = 7), followed by drug dispensation (including ARVs; n = 4).

Access to pediatric HIV testing and counseling is crucial for timely treatment to occur, especially given children's increased vulnerability to opportunistic infections (OIs) due to underdeveloped immune systems. HIV-positive children who have not been diagnosed are typically seen in clinics other than the HIV clinic for well visits, illnesses, or PMTCT related care. It is therefore important that pediatric testing is accessible in various clinics within a facility. Respondents were asked whether pediatric HIV testing was available in a variety of service areas at their site. Figure 2 shows their responses. The inpatient department was the only service identified by respondents at all sites as providing pediatric HIV testing.

**Figure 2. Services Offering Pediatric HIV Testing & Counseling**

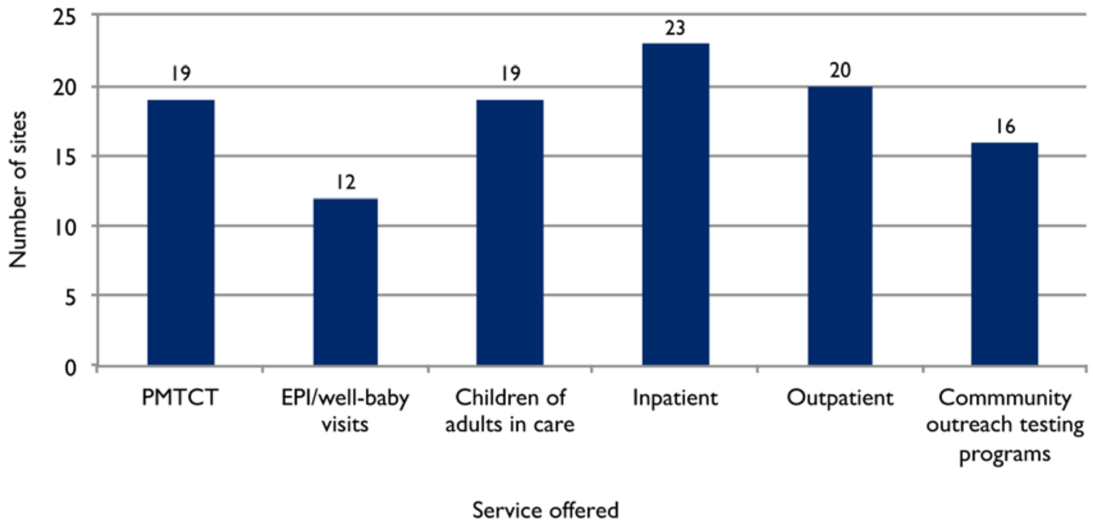


Respondents were also asked about the most common way that HIV-infected children enter into HIV care. The most common response (n = 18; 78%) was through PMTCT services (referral from antenatal clinic or labor and delivery).

# SERVICE INTEGRATION

Service integration has been the focus of efforts to simultaneously improve HIV-specific service delivery along with general health services. The necessity for some PLHIV to attend multiple facilities for various health care needs is often a barrier to optimal treatment outcomes. Sites were asked whether specific services were offered:

1. Within the pediatric HIV clinic
2. Onsite, but not within the pediatric HIV clinic
3. By referral only
4. Not available onsite or by referral.

Table 3 displays the number of sites offering each service in each location category. For those services accessed by referral to other sites, the referral process ranged from formal to informal by site. Services commonly offered within the pediatric HIV clinic were Integrated Management of Childhood Illness (IMCI) including malnutrition, HIV testing and counseling, ART adherence counseling, and phlebotomy. Services commonly offered onsite, but outside of the pediatric HIV clinic, were antenatal care, immunization, and family planning.  Values over 50 percent are highlighted within the table.

**Table 3. Service Integration**

| **Services** | **Number of sites with:** | | | |
|---|---|---|---|---|
| | ***Access within pediatric HIV clinic (%)*** | ***Access onsite, but not in pediatric HIV clinic (%)*** | ***Access within pediatric HIV clinic (%)*** | ***No access onsite or by referral (%)*** |
| Antenatal care (ANC) | 4 (17%) | 16 (70%) | 3 (13%) | - |
| Immunization (EPI) | 5 (22%) | 16 (70%) | 2 (9%) | - |
| Family planning | 6 (26%) | 16 (70%) | 1 (4%) | - |
| Integrated management of childhood illness (IMCI) including malnutrition | 17 (74%) | 6 (26%) | - | - |
| Inpatient | 9 (39%) | 14 (61%) | - | - |
| HIV testing & counseling | 15 (65%) | 8 (35%) | - | - |
| ART adherence counseling | 18 (78%) | 5 (22%) | - | - |
| Phlebotomy | 15 (65%) | 7 (30%) | 1 (4%) | - |
| TB treatment | 8 (35%) | 13 (57%) | 1 (4%) | 1 (4%) |
| Psychosocial support* | 14 (61%) | 7 (30%) | - | - |
| Linkages to other support services (i.e., housing, legal services)* | 9 (39%) | 7 (30%) | 4 (17%) | 2 (9%) |

* The total number of sites does not add up to 23 in these rows due to blank fields.

# ADOLESCENT SERVICES

Advances in pediatric HIV treatment have led to a growing number of perinatally infected children reaching adolescence. The growing number of adolescents living with HIV (ALHIV) has resulted in increased advocacy for this group and recognition of their unique care and treatment needs. Although WHO defines the adolescent age range as between 10 and 19 years of age, many sites base decisions on whether to transition pediatric patients to adult services on physical and cognitive development. The median reported upper age limit at sites for pediatric HIV programs was 14 (median range: 13–18).

Only two sites had established a specific clinic day for ALHIV. Eleven sites reported seeing adolescent patients in the pediatric clinic, whereas 16 saw them with adult ART patients. Sites were asked which of the five services in Table 4 were available and tailored to ALHIV. Those most commonly offered were tailored psychosocial support and positive prevention. According to data from the chart review, 45 percent (n = 27) of female pediatric ART patients aged 15 and older had received family planning counseling.

**Table 4. Services Tailored to Adolescents by Site**

| Services tailored to adolescents | Number of sites offering service (%) |
|---|---|
| Family planning and contraceptives | 12 (52%) |
| Psychosocial support | 18 (78%) |
| Peer support groups | 15 (65%) |
| Disclosure support | 14 (61%) |

When asked about the main challenges in treating adolescents, the most common answers were disclosure and nonadherence. Some adolescents have not been told about their HIV status by caregivers, despite the recommendation to begin the step-by-step disclosure process in perinatally infected children at the age of six (WHO 2011b). Adolescents who have not been disclosed to may suspect their positive status based on their daily medications and talk about HIV within their communities. "Accidental" disclosure has been associated with poor outcomes, and deteriorated relationships within the caregiver–patient unit. Engaging children and adolescents in a step-by-step disclosure process has been shown to improve ART adherence as patients often take increased ownership of their health (WHO 2011b). The issue of nonadherence among ALHIV may likely stem from a combination of other challenges that were mentioned. Respondents mentioned that many of their adolescent patients are vulnerable and/or orphans, often living in poverty without enough money for school materials or transport to appointments. Respondents also reported that adolescents sometimes become annoyed or dismissive when providers attempt to discuss safe sex.

Respondents at sites that were able to provide tailored services to adolescents reported on the benefits of connecting ALHIV through clubs and support groups and expressed pride at their clinic's ability to do so. Several also mentioned adolescent patients' involvement in HIV clubs within their schools. Support groups for caregivers of ALHIV were also provided at some clinics. Several respondents also discussed the scenario of adolescent patients bringing HIV-positive friends from outside of the clinic to support group meetings.

# HIV-EXPOSED INFANTS

For 19 (83%) of the sites, the PMTCT program was the most common way that HIV-exposed infants entered into HIV care. The second most common way was as children of adults in HIV care (n = 3). As discussed, PMTCT was also the most common way that HIV-infected children entered into HIV care. Approximately half the assessment sites (n = 11) reported following exposed infants within the HIV clinic. Seven sites followed them in the PMTCT clinic, whereas the remaining sites followed them at another clinic onsite.

The barrier to exposed infant care that was mentioned most often at facilities was the lack of money for caregivers to pay for opportunity costs[1] such as transport. Other patient-oriented challenges were that many HIV-positive women deliver offsite and/or bring their exposed children in once they are visibly ill and have missed several opportunities for prophylaxis. Loss to follow-up was another common issue for exposed infants, with caregivers sometimes not returning for dried blood spot (DBS) results. Finally, several providers mentioned maternal nonadherence to safe infant feeding plans and inadequate nutritional support from the clinic. Human resource constraints are a pervasive challenge that was mentioned in nearly each focus area. Providers mentioned the need for additional staff, and specifically additional staff trained in early infant diagnosis (EID). The frequent transfers of trained staff pose an ongoing challenge.

The majority of sites mentioned decreased mother-to-child transmission rates as their primary success in caring for exposed infants. As community members began to hear about the benefits of PMTCT, greater numbers of HIV-positive pregnant women began accessing the clinics and mother-to-child transmission rates fell even farther. Decreased mortality was another success shared with regard to EID. Two sites mentioned that HIV-positive women from the communities come to deliver at their facilities because they know about the comprehensive PMTCT services offered.

# ADHERENCE

All sites reported providing pediatric patients and caregivers with adherence support counseling prior to ART initiation. All but one site reported assessing adherence at every visit. Eighty-seven percent of pediatric ART patients sampled in the chart review had received adherence counseling at their last visit. Most sites (n = 21) reported that using patient/caregiver report and pill count (or measuring syrup) is an effective way to measure adherence. Laboratory tests are often used to help confirm nonadherence.

Providers reported a number of ways to deal with situations where nonadherence is suspected or detected. The most common strategy involved intense counseling of the caregiver to learn the reasons for nonadherence, reemphasizing the importance of adherence and counseling them on disclosure when applicable. Counsel also involved strategies for caregivers to employ such as using an alarm clock or treatment buddy for daily reminders or mixing the medication with food when suitable. Age-appropriate counseling for children is another method used to improve adherence.

An additional strategy was to decrease the time between patient drug pick-ups so that providers can more closely monitor patients. A number of sites used phone calls and home visits to provide

[1] An opportunity cost is the price of the next best thing you could have done if you had made a different choice. For example, although going to two separate sites to receive TB and HIV services may be the best choice for your health, this may require you to miss work, pay for childcare, and/or pay for transportation. These are the opportunity costs.

additional support to nonadherent patients. Providers sometimes referred patients and/or caregivers to support groups or individual peer support from organizations such as mothers2mothers.

Many of the adherence difficulties mentioned by providers were related to the inconvenience and discomfort of taking a daily (or twice daily) medication. Choosing a consistent time to take the pills can be challenging due to patients' school schedules and caregivers' work schedules. A busy social life for older children adds to the difficulty of finding a convenient medication time. Some children have several caregivers managing their medications which can lead to confusion over dispensing and dosing. Orphans may have a difficult time finding a caregiver or treatment buddy to share in the oversight of their treatment. Children sometimes find the taste of the medications unpleasant and may suffer from side effects such as nausea and vomiting. These factors contribute to some children refusing to take their daily medication and may be exacerbated if they do not know why they are taking it. Children who do know their status may be reluctant to take their medications due to external or self-stigma. Malnutrition and lack of nutritional support is another concern for those on HIV treatment. Providers should discuss any nutritional implications associated with a patient's regimen. At the facility level, some respondents stated the need for additional adherence counselors, space for adherence counseling, and posters offering adherence support.

# DEFAULTERS

The most commonly reported definition for loss to follow-up within the pediatric HIV clinics was "greater than three months late for the last scheduled appointment." The national pediatric treatment guidelines discuss loss to follow-up and contributing factors, but do not include a specific definition. All but one site reported having a system in place to identify pediatric HIV patients who had missed visits. Sites used either paper registers or a computer system to identify these patients. This task was often managed by the medical records or monitoring and evaluation departments. In terms of tracking patients, sites used a combination of phone calls and home visits (16 sites did home visits). Many sites had teams of specific staff members (e.g., Treatment Support Specialists) dedicated to this task.

The most common challenge reported in locating defaulters was caregivers providing incorrect contact information to facility staff. Caregivers who do provide accurate information sometimes deny their identity upon the outreach worker's arrival at the home. Respondents speculated that this may occur out of fear that home visits will disclose the caregiver or child's status to neighbors or unknowing members of the household. Some facilities lacked the ability to do home visits due to staff shortages. Several providers mentioned that they are not always made aware of patient deaths or relocation.

# TB/HIV CO-INFECTION

TB/HIV co-infection is a serious concern among providers and caregivers of pediatric patients. Children's underdeveloped immune systems coupled with HIV-related immunosuppression make HIV-positive children extremely vulnerable to TB. Twenty-one sites (91 percent) reported screening each pediatric HIV patient for TB at the initial visit to the HIV clinic. According to patient-level data from the chart review, 76 percent of pediatric HIV patients were screened for TB upon entry to care. Six sites reported treating TB/HIV co-infected pediatric patients within the pediatric HIV clinic and sixteen facilities were able to treat these patients onsite, but in a separate clinic. Nearly one-third of sites (n = 8) reported the use of isoniazid (INH) preventive therapy among TB-exposed pediatric HIV patients, as recommended in the guidelines. Most sites used chest X-rays and sputum

acid-fast bacillus (AFB) to test for TB. Facilities that lacked the proper equipment either referred exposed patients to another site or relied on history and a physical, and the TB symptoms checklist. A Score Chart for use as a Screening Tool for Tuberculosis in Children can be found in Annex I of the National Guidelines (FMoH 2010).

In terms of challenges, respondents noted difficulty when there was no "one-stop-shop" for HIV and TB services. In some cases, diagnosis and treatment were provided at separate locations—some onsite and some offsite. Diagnosis was another common issue—many sites lacked the equipment for this and young children have a difficult time producing the sputum necessary for cultures. A number of sites mentioned stock outs of pediatric TB drugs and many mentioned the difficulty in adjusting patients' ART regimens and initiating them on TB treatment. Pill burden is an issue for both patients and caregivers when a child is prescribed two daily regimens.

Respondents at sites that had not experienced stock outs of TB drugs pointed to drug availability as a success. Many providers stated improvements in patient health as a primary accomplishment of the TB program. Some TB clinics were co-located or integrated with HIV clinics. Successful referrals (both in and out) was another strength for some facilities.

# SUPPORTIVE SERVICES

Children living with HIV and their caregivers can greatly benefit from the supportive services offered within some clinics. Support groups (n = 22) and individual counseling (n = 21) were the most widely offered psychosocial support services, according to respondents. Family counseling was offered at 19 sites and disclosure counseling at 16 sites.

**Table 5. Psychosocial Support Services Offered by Site**

| Psychosocial support services | Number of sites offering service (%) |
|---|---|
| Disclosure support | 16 (70%) |
| Support groups | 22 (96%) |
| Family counseling | 19 (83%) |
| Individual counseling | 21 (91%) |

# NUTRITION

## GROWTH MONITORING

Nutrition and growth are crucial components of an HIV-positive child's overall well-being. The majority of sites reported growth monitoring at each visit. The most popular method of growth monitoring was weight- and height-for-age charts, which were available at all sites. Twenty-one sites also used dietary assessments, with 17 and 16 sites utilizing laboratory tests and head circumference, respectively.

**Table 6. Growth Monitoring Methods Used by Site**

| Growth monitoring methods | Number of sites using method (%) |
|---|---|
| Weight- and height-for-age charts | 23 (100%) |
| Head circumference | 16 (70%) |
| Mid-upper arm circumference | 4 (17%) |
| Skinfold test | 12 (52%) |
| Laboratory tests | 17 (74%) |
| Dietary assessment | 21 (91%) |

## FOOD SUPPORT, VITAMINS, AND MICRONUTRIENTS

Nineteen sites (83 percent) were able to provide some sort of food support to pediatric HIV patients. Five of those nineteen sites were able to provide additional food for caregivers. The most common type of food support reported was Plumpy'Nut.[2] Some sites were also able to provide other food such as vegetables, oil, rice, beans, milk, eggs, and crayfish. More than half of the sites providing food support reported stock outs, especially of Plumpy'Nut, lasting up to a year. Nearly all sites (n = 21) provided vitamins and/or micronutrients to pediatric HIV patients. Some sites prescribed these to every HIV-infected child whereas others determined eligibility through clinical assessment. Malnourishment, anemia, and OIs were some of the common eligibility criteria mentioned. Sites that do not provide vitamins and micronutrients to all pediatric HIV patients discontinue it once patients show marked improvements in physical examinations and laboratory tests, and once they reach their target weight. Occasional stock outs require some sites to temporarily stop providing vitamins.

[2]Plumpy'Nut is a peanut-based paste with a nutritional value equal to F-100 of therapeutic milk. It is used to aid in the nutritional rehabilitation of children suffering from severe acute malnutrition.

# COMMUNITY ENGAGEMENT

Many PLHIV find support within their communities. Linkages between HIV clinics and community-based organizations (CBOs) can help to bridge the gap between clinical care and psychosocial support. Eighteen sites reported partnerships with CBOs linking pediatric HIV patients to community services. The same number of sites reported partnerships with PLHIV groups that provided input into pediatric HIV services. Twenty-one sites reported receiving referrals from private practitioners for pediatric HIV patients. Finally, four sites had programs that engaged traditional healers.

Respondents mentioned that although these partnerships with CBOs exist, linkages between facilities and communities were minimal in practice. A number of sites pointed to a dearth of funding and staff as the limiting factors. A lack of community awareness and concern and stigma were other reasons mentioned.

For those facilities that had stronger linkages to their communities, respondents reported that community groups helped to create HIV awareness, which in turn, reduced stigma and left community members feeling more comfortable in seeking HIV-related services. Some CBOs provided nutritional and/or financial support through the provision of food, payment of school fees and/or transport, and sometimes escorting patients to appointments. Some CBOs have started support groups whereas others take an active role in reducing loss to follow-up through patient tracking. Finally, some providers felts that CBOs had played an important role in improving patient outcomes, including survival.

# PHARMACY

Pharmacy functionality within facilities is central to meeting patients' needs. Twenty-two site pharmacies (96 percent) each reported completing dispensing registers and using stock cards. All site pharmacies ordered pediatric HIV drugs and had security in place to protect the products. Nineteen sites (83 percent) were able to maintain cold chains and 17 sites (74 percent) had quality assurance measures in place. Eighteen sites were found to have adequate storage—including refrigeration space—for stocks of pediatric HIV drugs. Eleven of these sites reported having additional storage space available should the pediatric HIV program expand or if additional drugs required refrigeration in the future. To maintain drug stock, a median of two weeks was needed for pharmacies to receive an order of pediatric HIV drugs from the time the order was placed. Approximately half of sites (n = 12) required special approvals for second-line regimens or substitution pediatric ARVs.

All but one site reported a full stock of pediatric first-line regimens at the time of the assessment, although six had experienced stock outs of a first-line drug in the last quarter. The most commonly stocked out pediatric ARV was NVP syrup. Ten sites reported having current stocks of pediatric second-line regimens and three sites had experienced stock outs in the last quarter. Many of the 13 sites that did not have second-line regimens in stock reported that they had no pediatric patients requiring second-line therapy. Five sites reported stock outs of CTX in the last quarter. The same number reported PMTCT drug stock outs during that period. Four sites reported stock outs of pediatric phlebotomy supplies in the last quarter. Most sites (n = 16) did not have fluconazole for pediatric patients.

# LABORATORY

Laboratories are another critical component of the comprehensive care provided to children with HIV. Dysfunction within a lab can have serious consequences, such as delayed treatment, for those in care. Laboratories were open for an average of four days per week with a range of one day to seven days per week across sites. Three laboratories are open 24 hours per day, 7 days per week. All laboratories reported having a quality control program in place.

DBS turnaround time averaged 14.5 days across sites (median = 10.5 days), according to respondents. When asked about breaks in DBS testing or processing availability, respondents reported that there had been none. Sites reported the ability to offer rapid testing without any supply stock out issues. Eight sites were able to process viral loads whereas an additional two sites sent viral load samples to an offsite lab for processing. CD4 counts and percentages, complete blood counts, and liver function tests were performed at all but one site, with occasional equipment break downs. According to chart review data, 44 percent of patients alive and in care at the time of the chart review had a CD4 count or percent from the last six months in their charts. Both the 2007 and 2010 national pediatric guidelines recommend monthly CD4 monitoring for the first six months of treatment, followed by quarterly monitoring after six months.

The most common challenges mentioned by laboratory personnel were machine break downs, insufficient trained manpower, and an unsteady supply of reagents. In addition to reagents, approximately one-third of laboratory respondents mentioned other supplies lacking from their laboratories such as rapid tests, polymerase chain reaction (PCR) test kits, registers and forms, and microplate washers. Other challenges were an inadequate power supply and limited space within facilities. Respondents mentioned the difficulty of having to refer patients or send samples to other labs—often far away—for PCR and sometimes other tests, especially since turnaround times are often long and prohibit timely patient follow-up.

Although turnaround time for PCR and other tests may not be optimal, approximately one-third of laboratory respondents remarked that PCR turnaround time (TAT) had been decreasing, thereby leading to more timely treatment for those testing positive. Several mentioned that utilizing SMS to communicate results from the PCR lab to the clinic lab had reduced TAT even further. A few respondents reported that pediatric patients were prioritized in terms of laboratory services. Others mentioned that test results were accurate and reliable with few, if any, rejected samples. Personnel took pride in these statistics pointing out that improved laboratory services lead to improved treatment.

# EARLY INFANT DIAGNOSIS

EID enables providers to identify HIV-positive infants and initiate them on treatment immediately upon diagnosis. The high mortality rate of HIV-positive children younger than two years coupled with the 2010 national guidelines recommending ART initiation for all HIV-positive children younger than two make EID instrumental in the survival of these children. All but one site reported the ability to collect DBS samples for EID. Three sites began collecting DBS samples in 2006 whereas the remaining began between 2007 and 2011. Nurses were the most commonly trained cadre in DBS collection (n = 18 sites), followed by laboratory technicians (n = 12 sites) and medical officers (n = 10 sites). Exposed infants most commonly entered into care through the Inpatient Department (n = 22 sites) and the adult ART clinic (n = 21), presumably when their mothers were

being seen. EID is integrated into several clinics and services both within and across sites, the most common being the ART clinic (n = 21 sites).

**Figure 3. Entry Point for Exposed Infants and EID Integration**

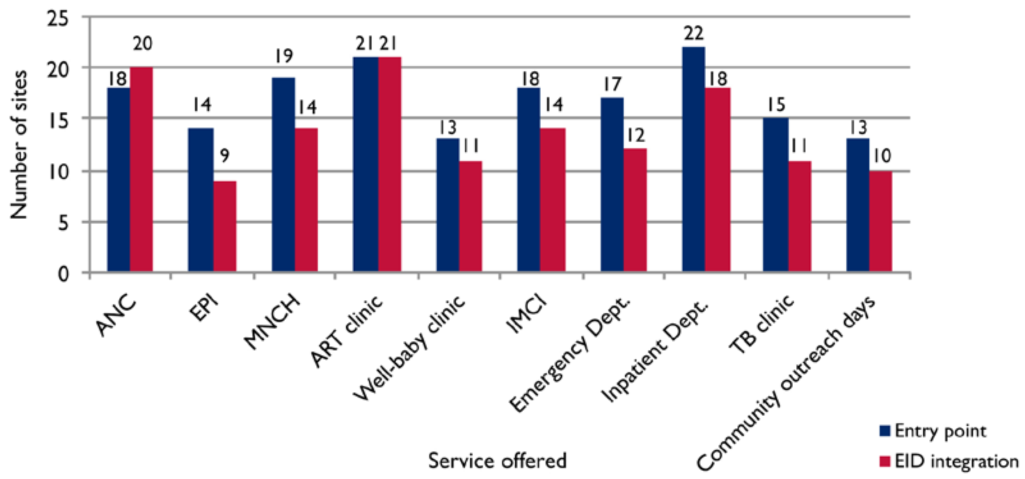


Most sites used a combination of methods to track exposed infants of HIV-positive women who had been seen during antenatal care (ANC) and/or delivery. Separate mother and baby registers and individual patient files were used most often (n = 15 sites each). Ten sites used a mother–baby follow-up register to track mothers and babies in the same place. The majority of sites (n = 20) reported that most HIV-positive mothers return to the clinic when their infant is six weeks of age for DBS. This is in line with the national guidelines. DBS collection was available an average of three days per week across sites. When a provider indicated that a child needed to have a DBS sample taken, most sites (n = 14) reported a wait time of under one hour for patients and caregivers. Others had to wait up to four hours, although not frequently. Eleven sites reported using rapid tests on HIV-exposed infants prior to DBS sample collection. This is recommended in the national guidelines if the child is between 9 and 18 months of age and it is at least six weeks post-breastfeeding cessations (FMoH 2010).

# WALK-THROUGH CHECKLIST

## BASIC RESOURCES

Data collectors observed the resources, tools, and layout of the 23 pediatric HIV clinics and asked staff members clarifying questions as needed. All sites had access to water, although to varying degrees. Approximately one-quarter of the sites had running water in all rooms. The same number used boreholes (i.e., wells). Three sites reported having running water in a limited number of rooms such as the laboratory or doctors' offices. Two sites had intermittent running water and the same number had to get water from other areas of the facility such as the general outpatient department (GOPD). One site said that they had to go outside of the facility for water. All sites were wired for electricity, although five reported issues such as an inadequate power supply or having no standby generator. Seven sites had clinic phones (i.e., mobile and/or landlines). Five used an intercom system and the remainder used personal cell phones or had attendants relay messages.

## DECISION AIDS

Sites had a variety of pediatric HIV decision aids displayed throughout the clinics. All but one site had pediatric ART initiation and dosing charts. Twenty sites each had pediatric clinical staging and cotrimoxazole dosing charts and 18 each had EID and pediatric ART regimen charts.

**Table 7. Decision Aids Available by Site**

| Decision aids | Number of sites with decision aid displayed (%) |
|---|---|
| Early infant diagnosis | 18 (78%) |
| Pediatric clinical staging | 20 (87%) |
| Pediatric ART initiation | 22 (96%) |
| Pediatric ART regimens | 18 (78%) |
| Pediatric ART dosing | 22 (96%) |
| Pediatric CTX dosing | 20 (87%) |
| Pediatric ART adherence | 14 (61%) |
| Pediatric defaulter outreach | 8 (35%) |
| Pediatric treatment failure management | 12 (52%) |
| Pediatric TB screening | 12 (52%) |
| Neurodevelopment | 5 (22%) |
| Nutrition | 15 (65%) |

## SUPPLIES

Data collectors completed a checklist of whether specific supplies or equipment were available within the pediatric HIV clinic at each site. Results are presented in Table 8.

**Table 8. Supplies/Equipment Available at Sites**

| Supplies/equipment | Number of sites with supply/equipment (%) |
|---|---|
| Scales for infants | 22 (96%) |
| Scales for older children | 23 (100%) |
| Tape measures for head circumference | 21 (91%) |
| Growth boards (for length/height) | 18 (78%) |
| Growth charts | 17 (74%) |
| Blood pressure cuffs in different sizes | 9 (39%) |
| Oxygen saturation machine | 2 (9%) |
| Stethoscope | 19 (83%) |

# PART I: OVERVIEW AND RECOMMENDATIONS

Although the challenges referenced in this report are not foreign to the FMoH, IPs, or other pediatric HIV stakeholders, this assessment lends a voice to health care providers on the front lines of providing HIV care and treatment to children living with HIV. Included here is an overview of the results discussed throughout Part I of this report and recommendations to address the most common barriers to providing quality pediatric HIV treatment services.

## HUMAN RESOURCES

Many respondents mentioned the standard reassignment of trained personnel as the most debilitating human resources (HR) challenge facing their clinics. Although staff shortages are common among health care systems in low-income countries facing a high HIV burden, many respondents felt that limiting the number of trained personnel who were transferred to other sites would go a long way in mitigating HR challenges, allowing facilities to better serve their patients.

## CAREGIVERS

The substandard care that some children living with HIV receive from their caregivers is a great concern to health care providers and is often a manifestation of caregiver stress. Provider concerns around caregiving were especially common in cases of loss to follow-up—most notably among pre-ART patients—and in caregivers providing inaccurate addresses and phone numbers to avoid follow-up—perhaps due to a fear of stigma. Instead of this leaving caregivers and providers at odds with one another, it can be viewed as an unmet need for additional caregiver support. Caregivers need additional guidance around the importance of bringing the children they care for in for regular appointments, instead of waiting until they are ill. They also need to feel supported instead of judged. Similar to the mentor mother model initiated by mothers2mothers, AIDSTAR-One suggests a mentor caregiver initiative whereby attentive (nonparental) caregivers mentor those who are struggling, through psychosocial support and advice.

## DISCLOSURE

Beginning the disclosure process with an HIV-positive child is an overwhelming task for caregivers. Although there is increasing guidance on this issue from some of the leaders in HIV policy, such as WHO, providers and caregivers carry this burden. One helpful resource for providers is included in the International Center for AIDS Care and Treatment Programs' "Adolescent HIV Care and Treatment: A Training Curriculum for Multidisciplinary Health Care Teams." Module 7 is on "Providing Disclosure Counseling and Support." This document is available at: http://www.columbia-icap.org/resources/supporttools/index.html.

For disclosing to younger children, AIDSTAR-One is working with South to South and the François-Xavier Bagnoud Center (FXB) to adapt three booklets to guide caregivers through the step-by-step disclosure process with HIV-positive children. AIDSTAR-One plans to offer these to USAID/Nigeria for dissemination if deemed suitable by the FMoH.

# ADHERENCE

Good adherence is very closely linked to the two preceding subjects: caregivers and disclosure. Young children depend on their caregivers to pick up their medications from the pharmacy and to remind them to take their medication on a daily basis. Adolescents may have more autonomy with their medication management, but typically rely on caregivers to some extent and may become suspicious or resentful of their daily medication if they have not been disclosed to yet. Children at various ages can be provided with pill calendars and can be encouraged to personalize them or make their own. This is an inexpensive way for children and adolescents living with HIV to learn medication management. The use of pill boxes is another way to make good adherence achievable for patients. If no pill boxes are available onsite, patients and caregivers with means to buy their own should be encouraged to do so. Site personnel may also want to inquire with their IPs or donors as to whether pill boxes can be provided.

# ADOLESCENTS

Respondents were especially concerned for their adolescent patients, many of whom are OVCs. With the increasing population of adolescents living with HIV, more clinics should consider targeting services to this group. Nonadherence and other threats to good health can be exacerbated during this time of increased independence and free will. Two of the assessment sites did have targeted programs in place for ALHIV. AIDSTAR-One recommends information sharing between clinics with well-established ALHIV programs and those interested in starting one. In addition to the three disclosure booklets mentioned above, AIDSTAR-One is working with Teen Club Botswana to adapt "Teen Talk," a comprehensive question and answer guide for adolescents living with HIV, for use among ALHIV in other countries. This is another document that will be offered to USAID/Nigeria for dissemination. The guide was created hand-in-hand with ALHIV who participate in Teen Club Botswana, which is an impressive model aimed at empowering ALHIV that may be worth replicating in Nigeria. Finally, AIDSTAR-One has created an Adolescent Toolkit for providers of ALHIV, which will be posted on the AIDSTAR-One website in the coming months. Both Teen Talk and the Toolkit address hard-hitting adolescent issues such as disclosure, dating, and safe sex. They also provide guidance on psychosocial support that is targeted to ALHIV, such as peer support groups.

# DATA MANAGEMENT

Inadequate data management systems impede providers' abilities to properly care for patients. There is valuable time lost locating patient files, searching for missing information, and repeating services (e.g., laboratory tests) that have not been properly recorded. Patients suffer when providers are unable to make informed treatment decisions due to missing information. Similarly, when aggregate site-level data is inaccurate, program managers and FMoH officials are unable to identify important trends and areas for improvement. Continued technical assistance and capacity building in this area will serve to improve quality of care. Twelve assessment sites (52 percent) currently use EMRs to manage patient data. Implementing partners and/or individual sites that are successfully managing

patient data electronically should be asked to share best practices in EMR training and implementation with the FMoH for use with other IPs/sites that are ready to make the transition to EMR. Providers appreciate the aggregate data that EMR systems are able to generate, allowing them to see trends in patient care and outcomes. The successful expansion of EMRs across more sites would also serve to improve data collected at the national level.

# SITE RESOURCES

Providing quality care to children living with HIV requires a wide variety of resources and support systems at the site level. One area that sites reported doing well in was nutrition. Although malnutrition was a big provider concern, especially among orphans and vulnerable children (OVC), 83 percent of sites were able to provide food support to patients. Updated guidance on breastfeeding in the national guidelines also helped to improve nutrition among young patients. Sites, however, were not able to meet other patient needs as successfully. Many lacked basic resources such as reliable electricity and easily accessible water. Lab personnel reported machine break downs and an unsteady supply of reagents. Although there are no inexpensive or quick fixes to these issues, especially given the number of treatment facilities in such a large country, they need to be addressed both for sake of PLHIV and the morale of the providers who care for them.

# PART II: METHODOLOGY, RESULTS, AND CONCLUSION

Data from a number of observational datasets throughout sub-Saharan Africa have demonstrated significant benefits in morbidity and mortality from treatment with ART in children and adolescents (Fenner et al. 2000; Wamalwa et al. 2010; Callens et al. 2009; Zanoni et al. 2011; Kabue et al. 2012). Although efforts have been underway in Nigeria to increase access to ART for pediatric patients since 2005, limited data exist describing treatment outcomes in this population. Data recently presented noted that in a cohort of pediatric patients (age < 14) treated with ART for a mean duration of 21 months at clinics supported by a single implementing partner in Nigeria, 67.5 percent were found to be virally suppressed (Mwangi et al. 2012). To date, no data have been published documenting pediatric and adolescent outcomes from a variety of different treatment settings and facility types.

Providing high quality care and treatment to this population continues to be challenging in part due to health systems deficits. Inadequately trained health care workers, insufficient pediatric formularies, lack of laboratory facilities, as well as many other factors endemic to resource limited settings have slowed pediatric scale-up (Kline 2006). As ART access for children and adolescents increases, determining whether or not the health system factors contribute to outcomes for patients who are in care and accessing treatment is important. Increased understanding of the facility level characteristics that are associated with outcomes may help guide future programmatic planning.

Part I of this report details findings from a site assessment survey administered at 23 sites providing pediatric care and treatment. Information gathered is described in Part I. In order to provide an overview of clinical outcomes of the children and adolescents treated at these sites and to explore the impact of these health system characteristics on outcomes and quality of care, AIDSTAR-One conducted the following study.

## METHODOLOGY

### SETTING

Approximately 35,900 children and adolescents ages have been initiated on ART through the Nigerian National HIV Treatment Program since its inception in 2002 through December 2011. Pediatric care and treatment is provided at public, tertiary care, and district level hospitals throughout the country. Funding for treatment is provided by the Global Fund to Fight AIDS, TB and Malaria and the FMoH.

## STUDY DESIGN

This is a retrospective cohort study covering the time period of November 2002 to December 2012. Patients were eligible for inclusion in the study if they initiated ART during this time period and were < 19 years of age. Age younger than 19 was chosen as an upper limit cut off to incorporate adolescents as defined by WHO.

Data was captured from all 23 sites included in Part I of this assessment. Paper medical charts were utilized at 19 of the 23 sites that were included in this study. The remaining four sites utilized EMRs and data was extracted directly from the electronic record. Collected clinical data included age, gender, date of enrollment in care, date of first positive HIV test, date of ART initiation, baseline CD4 count and percentage and subsequent immunologic data (obtained every six months per guidelines), ART regimen, WHO stage at enrollment, and baseline weight/height. Quality data such as evidence of screening for TB at initial visit, adherence counseling at last visit, and family planning counseling in female patients age 15 and older were also collected.

Loss to follow-up was defined as no evidence of a visit to the clinic or drug pick up for 3 months following the patient's last visit (or 90 days following the most recent missed scheduled visit) as documented in the chart. Death and transfers were documented as recorded in the patient's chart. Cause of death was not captured. All medical records included a field indicating that the patient had been contacted to confirm death, loss to follow-up, and transfers. If this field was checked, death, loss to follow-up, and transfers were considered confirmed. All loss to follow-ups and transfers were censored on the date of their last visit. Otherwise follow-up time was censored at the end of the study period.

Data was entered into an Access database specifically designed for this study. Unique patient identifiers were assigned to each patient to protect patient confidentiality.

## SAMPLING

Systematic random sampling was used to select a designated number of charts for review at each site. Each implementing partner was asked to provide a list of all enrolled pediatric patients meeting the inclusion criteria by medical record number. Based on the total number at each site every nth record on that list was selected for review. For example, if the total number of enrolled available patients at a site was less than 280, then every third chart was reviewed. If the total number of patients at a site was between 281 and 400, then every fourth chart was reviewed and so on. Charts missing portions of clinical data were included in this review.

## DATA ANALYSIS

We performed a series of data analyses starting with the generation of descriptive statistics for all salient variables. We computed means, standard deviations, and quintiles for continuous variables and counts with percentages for categorical variables. Next, we used bivariate methods to examine the relationships of individual independent variables with our primary outcomes of survival and loss to follow-up. The variables of interest included child gender, age at ART initiation, CD4 count at ART initiation, the z-score for the child's body weight at ART initiation (based on U.S. Centers for Disease and Prevention [CDC] norms), time delay in ART treatment, the type of initial ART treatment, the use of second-line ARV, the application of Nigerian national guidelines for ART, a score summarizing the degree of service integration, the location (rural, urban) and type (primary,

secondary, tertiary) of the treating hospital, and the number of part- and full-time hospital staff. These analyses were conducted using Kaplan-Meier estimation with log rank testing and one predictor Cox proportional hazards regression models for survival as well as cross-tabulations with chi-square tests and Fisher Exact tests or one predictor logistic regression models for loss to follow-up.

In our survival analyses, associations were estimated using hazard ratios with 95 percent confidence intervals (CI). In the analyses of loss to follow-up, we estimated associations using odds ratios with 95 percent CIs. For each outcome of interest, we then constructed multivariable regression models with the set of independent variables that were examined in the bivariate analyses. The proportional hazards assumptions were checked by graphical methods and by testing interaction terms that included the log of follow-up time. The discrimination ability of the logistic models was measured by c-statistics with calibration assessed using Hosmer-Lemeshow chi-square statistics and their associated P values. Where data were sufficient, we tested for salient interactions among the independent variables in these models—for example, with gender, hospital type, and initial CD4 category. We employed an alpha of 0.05 in all statistical tests to determine statistical significance. All data management and statistical analyses were performed using SAS for Windows version 9.2.

# RESULTS

## BASELINE CHARACTERISTICS

There were 1,516 children sampled from 23 sites. Baseline characteristics are summarized in Table 9. Overall, mean age at initiation was 64 months (SD 51.7). Severe immunosuppression (see Appendix A) was noted in 45 percent (n = 653) at enrollment. Mean z-score was –1.1 (SD 4.0). Median duration of follow-up was 24.4 months. Median time from ART eligibility to ART initiation was 7.7 weeks. The most common initial regimen was AZT/3TC/NVP (81.5 percent). Forty-two percent of patients experienced a delay to ART initiation of greater than 12 weeks from enrollment. Please see Appendix D for baseline characteristics by site.

**Table 9. Characteristics of Sampled Pediatric and Adolescent Patients in Nigerian Treatment Program**

| | Number | |
|---|---|---|
| **Total sample** | 1516 | |
| ***Demographics*** | | |
| Mean age at initiation in months (SD) | 1511 | 64.4 (±51.7) |
| Mean age at initiation by group (SD) | | |
| 0–24 months | 363 | 14.1 (±6.2) |
| 25–71 months | 605 | 43.4 (±13.9) |
| 6–9 years | 318 | 7.8 (±1.1) |
| 10 years or older | 225 | 13.4 (±3.0) |
| Gender (Male, %) | 799 | 52.8 |
| | | |
| ***Clinical Factors*** | | |
| Initial CD4 count[a] (SD) | 1357 | 672.4 (±603.1) |
| Initial CD4 percentage[b] (SD) | 512 | 19.9 (±21.0) |
| Severe immunosuppression[c] (%) | 653 | 45.4 |
| Moderate immunosuppression[d] (%) | 475 | 33.0 |
| Mean weight for age (z-score) (SD) | 1382 | –1.08 (±4.04) |
| | | |
| ***Treatment*** | | |
| Mean time from eligibility to ART initiation in weeks (SD) | 1255 | 26.2 (±43.0) |
| Median time from eligibility to ART initiation in weeks | 1255 | 7.7 |
| Mean duration of follow-up in months (SD) | 1511 | 27.7 (±19.7) |
| Median duration of follow-up in months | 1511 | 24.4 |
| ***Current ART regimens*** | | |
| AZT/3TC/NVP (%) | 1236 | 81.5 |
| AZT/3TC/EFV over 3 years of age at initiation (%) | 88 | 6.7 |
| Regimens containing EFV (%) | 110 | 7.3 |
| Regimens containing d4T (%) | 81 | 5.3 |
| Mean length of time on d4T containing regimen in months (SD) | 69 | 36.7 (±18.5) |
| Regimens containing LPV/r (%) | 53 | 3.5 |

[a, b, c, d] refer to Appendix A.

## QUALITY DATA

Well-validated indicators of quality of HIV care were also collected. Most patients were screened for TB at entry into care (76.3 percent) and had adherence counseling at their last visit (86.9 percent). Less than half of the female patients age 15 years and older received family planning counseling (45 percent). Almost all patients had been prescribed CTX at some point (98.5 percent). Less than half of patients alive and in care at the time of the chart review had a CD4 count in the six months prior to chart review (44 percent) (Table 10).

**Table 10. Quality Data**

| | **Number (%)** |
|---|---|
| Percent screened for TB at entry into care | 1115 (76.3%) |
| Percent with adherence counseling documented at last visit | 1311 (86.9%) |
| Percent of female patients 15 or older who received family planning counseling | 27 (45.0%) |
| Percent ever prescribed CTX | 1482 (98.5%) |
| Percent of patients alive and not loss to follow-up with at least one CD4 count in last six months | 487 (44.0%) |

No associations were observed between age categories and screening for TB at entry into care (P = 0.2332), adherence counseling (P = 0.2608), or ever prescribed CTX (P = 0.4098). No associations were observed between immunosuppression and screening for TB (P = 0.0597) or ever prescribed CTX (P = 0.4866) (Table 11).

**Table 11. Bivariate Analysis of Immunosuppression and Quality Indicators**

| | **Immunosuppression** | | | |
|---|---|---|---|---|
| | ***Severe Number (%)*** | ***Moderate Number (%)*** | ***No Suppression Number (%)*** | ***P value*** |
| ***Percent screened for TB at entry into care*** | 471 (73.9%) | 361 (79.2%) | 232 (77.9%) | 0.0597 |
| ***Percent with adherence counseling documented at last visit*** | 569 (87.4%) | 399 (84.4%) | 284 (91.3%) | 0.0031 |
| ***Percent ever prescribed*** | 642 (99.1%) | 463 (98.1%) | 305 (98.4%) | 0.4866 |

## MORTALITY AND LOSS TO FOLLOW-UP

Mortality within 90 days of ART initiation was 1.9 percent. A total of 4.2 percent of patients died during the period of follow-up (mean 27.7 months). Loss to follow-up was 19.1 percent during the follow-up period (Table 12).

**Table 12. Outcomes from the Pediatric HIV Treatment Program**

| | **Number (%)** |
|---|---|
| Mortality | |
| Within 90 days of ART initiation | 30 (1.9%) |
| During period of follow-up (mean 27.7 months) | 64 (4.2%) |
| | |
| Loss to Follow-Up | |
| Within 6 months of ART initiation | 52 (3.6%) |
| Within 12 months of ART initiation | 100 (6.9%) |

## FACTORS ASSOCIATED WITH MORTALITY AND LOSS TO FOLLOW-UP

We sought to determine whether or not associations existed between a number of variables and both loss to follow-up and mortality. In bivariate results, weight for age z-score (HR: 0.9; 95% CI: 0.8–0.9), moderate immunosuppression (HR: 7.2; 95% CI: 1.4–77.4), severe immunosuppression (HR: 9.7; 95% CI: 1.3–72.6), and enrollment at a rural health center (HR: 10.2; 95% CI: 3.2–32.3) were associated with mortality. In multivariate results, adjusting for other factors, both moderate immunosuppression (AHR: 8.6; 95% CI: 1.1–66.4) and severe immunosuppression (AHR: 9.0: 95% CI: 1.2–68.4) were significant predictors of mortality, along with enrollment at a rural health center (AHR: 24.2; 95% CI: 2.3, 255.1) (Table 13).

In regard to loss to follow-up, in bivariate results, moderate immunosuppression (OR: 1.6; 95% CI: 1.0–2.6), severe immunosuppression (OR: 1.6; 95% CI: 1.0–2.9), age younger than 2 years (OR: 1.8; 95% CI: 1.2–2.6) were associated with loss to follow-up. In multivariate results, adjusting for other factors, age younger than 2 years (AOR: 1.6; 95% CI: 1.1–2.4) was independently associated with loss to follow-up (Table 13). Loss to follow-up was not associated with any other patient characteristics or site level factors.

**Table 13. Factors Associated with Death and Loss to Follow-Up**

| | Bivariate Correlations with Mortality HR (95% CI) | Bivariate Correlations with Loss to Follow-Up OR (95% CI) | Multivariate Model for Mortality AHR (95% CI) | Multivariate Model for Loss to Follow-Up AOR (95% CI) |
|---|---|---|---|---|
| Weight for age z-score | 0.9 (0.8, 0.9) | 1.0 (0.9, 1.1) | 0.92 (0.84, 1.0) | 1.0 (0.9, 1.1) |
| Baseline level of immunosuppression | | | | |
| No suppression | *Ref* | *Ref* | *Ref* | *Ref* |
| Severe | 9.7 (1.3, 72.6) | 1.6 (1.0, 2.9) | 9.0 (1.2, 68.4) | 1.4 (0.9, 2.2) |
| Moderate | 10.2 (1.4, 77.4) 7777.77777.4) 77.4) | 1.6 (1.0, 2.6) | 8.6 (1.1, 66.4) | 1.5 (0.9, 2.4) |
| Age | | | | |
| 0–24 months | 1.4 (0.6, 3.3) | 1.8 (1.2, 2.6) | 1.4 (0.6, 3.3) | 1.6 (1.1, 2.4) |
| 25–71 months | *Ref* | *Ref* | *Ref* | *Ref* |
| 6-9 years | 1.4 (0.6, 3.4) | 1.0 (0.6, 1.6) | 1.5 (0.6, 3.6) | 1.1 (0.7, 1.7) |
| 10 years or older | 1.7 (0.6, 4.9) | 1.1 (0.6, 2.0) | 1.0 (0.3, 3.6) | 1.1 (0.6, 1.9) |
| Gender (Male) | 1.3 (0.7, 2.6) | 1.0 (0.7, 1.4) | 1.3 (0.6, 2.5) | 1.0 (0.7, 1.4) |
| Use of 2010 guidelines | | | | |
| Fully | 0.8 (0.4, 2.0) | 1.0 (0.6, 1.5) | 1.3 (0.5, 3.1) | 1.1 (0.7, 1.8) |
| Partially | 1.8 (0.6, 5.5) | 0.4 (0.7, 2.7) | 0.5 (0.1, 3.7) | 2.0 (1.0, 3.9) |
| None | *Ref* | *Ref* | *Ref* | *Ref* |
| Site type | | | | |
| Tertiary hospital | *Ref* | *Ref* | *Ref* | *Ref* |
| Secondary hospital | 1.4 (0.6, 3.2) | 1.1 (0.7, 1.6) | 1.6 (0.7, 3.9) | 1.1 (0.7, 1.6) |
| Primary hospital | 0.0 (0.0, -- ) | 1.5 (0.6, 3.8) | 0.0 (0.0, -- ) | 1.6 (0.6, 4.3) |
| Rural health center | 10.2 (3.2, 32.3) | 0.6 (0.1, 2.8) | 24.2 (2.3, 255.1) | 0.3 (0.1, 1.8) |

# RESULTS SUMMARY

- The retrospective chart review provides data on more than 1,500 systematically sampled pediatric and adolescent patients enrolled at 23 different treatment sites from 10 states throughout Nigeria.
- Nearly half (45 percent) of all patients presented with severe immunosuppression at enrollment.
- Forty-two percent experienced a delay from enrollment (first HIV positive test) to ART initiation of more than 12 weeks.

- Most patients were screened for TB at entry into care (76.3 percent) and received adherence counseling at their last visit (86.9 percent).
- Less than half of patients alive and in care at the time of the chart review had a CD4 count documented in their chart in the six months prior to chart review (44 percent).
- Mortality during the period of follow-up (mean 27.7 months) was 4.2%.
- Loss to follow-up was 19 percent during the follow-up period.
- Moderate immunosuppression and severe immunosuppression at presentation were significant predictors of mortality.
- Enrollment at a rural health center was independently associated with mortality. Additional analysis is warranted to further understand this finding.
- Moderate immunosuppression, severe immunosuppression, and age younger than two years (OR: 1.8; 95% CI: 1.2–2.6) were independently associated with loss to follow-up.
- Loss to follow-up was not associated with any other patient characteristics or measured site level factors.

# CONCLUSION

As pediatric treatment scale-up has progressed in Nigeria, many challenges have emerged.

Heavy patient loads, staff shortages, and inadequate resources are key challenges noted in this report. However, providers surveyed for this assessment noted the improved health outcomes among their patients thanks to increased access to ART with pride. Many provided HIV care when there were no treatment options for HIV-positive children and expressed that they are inspired by the improved prognosis for these children over the past decade. Nigeria has made notable progress toward decreasing HIV-related morbidity and mortality among children living with HIV throughout the country. Our data show that treatment outcomes are encouraging. However, the need for interventions to ensure earlier access to ART and decrease loss to follow-up is evident. By addressing the challenges mentioned in this report and continued prioritization of the provision of high quality HIV care, Nigeria will continue to note an increasing number of children living with HIV reach adolescence and adulthood.

# REFERENCES


Callens, SF, N Shabani, J Lusiama, et al. 2009. "Mortality and Associated Factors After Initiation of Pediatric Antiretroviral Treatment in the Democratic Republic of the Congo." *The Pediatric Infectious Disease Journal* 28:35-40.

Federal Ministry of Health Nigeria (FMoH). 2010. *National Guidelines on Paediatric HIV and AIDS Treatment and Care*. Abuja: FMoH.

Fenner, L, MW Brinkhof, O Keiser, et al. 2010. "Early Mortality and Loss to Follow-up in HIV-infected Children Starting Antiretroviral Therapy in Southern Africa." *Journal of Acquired Immune Deficiency Syndromes* 54:524-32.

Joint United Nations Programme on HIV/AIDS (UNAIDS). 2010. *Global Report: UNAIDS Report on the Global AIDS Epidemic 2010*. Geneva: UNAIDS. Available at http://www.unaids.org/globalreport/documents/20101123_GlobalReport_full_en.pdf

Kabue, MM, WC Buck, SR Wanless, et al. 2012. "Mortality and Clinical Outcomes in HIV-Infected Children on Antiretroviral Therapy in Malawi, Lesotho, and Swaziland." *Pediatrics* 130(3):e591-9.

Kline, MW. 2006. "Perspectives on the Pediatric HIV/AIDS Pandemic: Catalyzing Access of Children to Care and Treatment." *Pediatrics* 117(4):1388-93.

Mwangi, I, A Amoroso, D Watson, et al. 2012. *Virologic Outcomes of First-line Antiretroviral Treatment in HIV Infected Children in AIDSRelief Clinics in Nigeria*. International AIDS Society Meeting: AIDS 2012. Abstract no. MOPE043.

United Nations Millennium Development Goals (UN MDGs). 2011. "Goal 6: Combat HIV/AIDS, Malaria and other diseases." Available at www.un.org/millenniumgoals/aids.shtml

Wamalwa, DC, EM Obimbo, C Farquhar C, et al. 2010. "Predictors of Mortality in HIV-1 Infected Children on Antiretroviral Therapy in Kenya: A Prospective Cohort." BMC *Pediatrics* 10:33.

World Health Organization (WHO). 2010. *Antiretroviral Therapy for HIV Infection in Infants and Children: Towards Universal Access: Recommendations for a Public Health Approach - 2010 revision.* Geneva: WHO. Available at http://www.who.int/hiv/pub/paediatric/infants2010/en/index.html

World Health Organization (WHO). 2011a. *Global HIV/AIDS Response: Epidemic Update and Health Sector Progress Towards Universal Access: Progress Report 2011*. Geneva: WHO. Available at http://www.who.int/hiv/pub/progress_report2011/en/

World Health Organization (WHO). 2011b. *Guideline on HIV Disclosure Counseling for Children up to 12 Years of Age*. Geneva: WHO. Available at http://whqlibdoc.who.int/publications/2011/9789241502863_eng.pdf

Zanoni, BC, T Phungula, HM Zanoni, et al. 2011. "Risk Factors Associated with Increased Mortality among HIV Infected Children Initiating Antiretroviral Therapy (ART) in South Africa." *PLoS One* 6(7):e22706.

# APPENDIX A

# IMMUNOSUPPRESSION CATEGORIES

**Patients < 2 years of age:**

Severe immunosuppression: initial CD4 count less than 750 cells/mm3 or an initial CD4 percentage less than 15 percent.

Moderate immunosuppression: initial CD4 count of between 750 and 1500 cells/mm3 or an initial CD4 percentage of between 15 and 25 percent.

No immunosuppression: initial CD4 count of 1500/mm3 or more, or a CD4 percentage of 25 percent or more.

**Patients between 2 and 5 years of age:**

Severe immunosuppression: initial CD4 count less than 500 cells/mm3 or an initial CD4 percentage between 15 percent.

Moderate immunosuppression: initial CD4 count between 500 and 1000 cells/mm3 or an initial CD4 percentage between 15 and 25 percent.

No immunosuppression: initial CD4 count of 1000 cells/mm3 or more, or an initial CD4 percentage of 25 percent or more.

**Patients 5 years of age and older:**

Severe immunosuppression: initial CD4 count less than 200 cells/mm3 or an initial CD4 percentage less than 15 percent.

Moderate immunosuppression: initial CD4 count between 200 and 500 cells/mm3 or an initial CD4 percentage between 15 and 25 percent.

No immunosuppression: initial CD4 count of 500 cells/mm3 or more, or an initial CD4 percentage of 25percent or more.

# APPENDIX B

# PEDIATRIC ART INITIATION CRITERIA AND REGIMENS, NATIONAL GUIDELINES 2010

| ART Eligibility Criteria | First-line Regimens | Second-line Regimens | Third-line Regimens |
|---|---|---|---|
| All children < 24 months<br><br>Children 24–59 months with:<br>• CD4% ≤ 25% or CD4 count ≤ 750 cells/mm3; or<br>• WHO pediatric clinical stage 3 or 4<br><br>Children ≥ 5 years with:<br>• CD4 < 350 cells/mm3; or<br>• WHO pediatric clinical stage 3 or 4 | Children < 2 years with no prior NNRTI exposure:<br>Preferred:<br>AZT + 3TC + NVP<br><br>Alternatives:<br>ABC + 3TC + NVP;<br>AZT + 3TC + ABC; *or*<br>d4T + 3TC + NVP<br><br>Children < 12 months with prior NNRTI exposure:<br>Preferred:<br>AZT + 3TC + LPV/r<br><br>Alternatives:<br>ABC + 3TC + LPV/r; *or*<br>AZT + 3TC + ABC<br><br>Children 12 months to 2 years with NNRTI exposure:<br>Preferred:<br>AZT + 3TC + LPV/r<br><br>Alternatives:<br>AZT + 3TC + ABC;<br>ABC + 3TC + LPV/r; *or*<br>d4T + 3TC + LPV/r | If failed on AZT + 3TC + NVP:<br>ABC + 3TC + LVP/r; *or*<br>d4T + 3TC + LPV/r<br><br>If failed on ABC + 3TC + NVP:<br>AZT + 3TC + LVP/r;<br>d4T + 3TC + LPV/r; *or*<br>ddI + 3TC + LPV/r<br><br>If failed on AZT + 3TC + ABC:<br>ddI + 3TC + NVP;<br>TDF + 3TC + EFV; *or*<br>ddI + 3TC + LPV/r<br><br>If failed on d4T + 3TC + NVP:<br>ABC + 3TC + LVP/r;<br>ddI + 3TC + LPV/r; *or*<br>AZT + 3TC + ABC<br><br>If failed on AZT + 3TC + LPV/r:<br>ABC + 3TC + ddI; *or*<br>ddI + ABC + NVP *or* EFV<br><br>If failed on ABC + 3TC + LPV/r:<br>AZT + 3TC + ddI; *or* | Children with such needs should be referred to higher levels of care or ART specialists if necessary.<br>When salvage treatment is unavailable, a second-line failing regimen may be continued as there is evidence of some benefit despite emergence of resistance mutations. |

| | | | |
|---|---|---|---|
| | Children 2–3 years, regardless of NNRTI exposure:<br>Preferred:<br>AZT + 3TC + NVP<br>Alternatives:<br>ABC + 3TC + NVP;<br>AZT + 3TC + ABC; *or*<br>d4T + 3TC + NVP<br>Children > 3 years:<br>Preferred:<br>AZT + 3TC + EFV<br>Alternatives:<br>AZT + 3TC + NVP;<br>AZT + 3TC + ABC;<br>d4T + 3TC + NVP; *or*<br>ABC + 3TC + EFV | AZT + 3TC + NVP *or* EFV<br><br>If failed on AZT *or* d4T + 3TC + EFV:<br>ABC + 3TC + LPV/r<br><br>If failed on TDF + 3TC or FTC + EFV *or* NVP:<br>AZT + 3TC + LVP/r;<br>d4T + 3TC + LPV/r;<br>ABC + 3TC + AZT; *or*<br>ABC + 3TC + d4T | |

# APPENDIX C

# SAMPLE OF TWENTY-THREE SELECTED FACILITIES

| State | LGA | Site | Site Type | Setting | Implementing Partner |
|---|---|---|---|---|---|
| Enugu | Enugu East | Annunciation Specialist Hospital, Eneme | Secondary Hospital | Urban | AIDSRelief |
| | Awgu | University of Nigeria Teaching Hospital, Ozalla | Tertiary Hospital | Rural | AIDS Prevention Initiative in Nigeria /Harvard |
| | Udi | Udi District Hospital, Udi | Primary Hospital | Rural | Global HIV/AIDS Initiative Nigeria/FHI |
| Akwa Ibom | Uyo | University of Uyo Teaching Hospital, Uyo | Tertiary Hospital | Urban | Institute of Human Virology Nigeria |
| Cross River | Calabar Municipal | General Hospital Calabar | Secondary Hospital | Urban | Global HIV/AIDS Initiative Nigeria/FHI |
| | Ogoja | General Hospital, Ogoja | Secondary Hospital | Urban | International Center for AIDS Care and Treatment Programs |
| Taraba | Jalingo | State Specialist Hospital, Jalingo | Tertiary Hospital | Urban | Management Sciences for Health Prevention and Organizational Systems AIDS Care and Treatment |
| Niger | Bida | General Hospital, Bida | Secondary Hospital | Rural | Management Sciences for Health Prevention and Organizational Systems AIDS Care and Treatment |
| | Lapai | General Hospital, Lapai | Primary Hospital | Rural | Management Sciences for Health Prevention and Organizational Systems AIDS Care and Treatment |
| Benue | Ukum | NSKT Hospital, Zaki Biam | Secondary Hospital | Peri-urban | Christian Health Association of Nigeria |
| | Gboko | General Hospital, | Secondary | Urban | Global HIV/AIDS |

| | | | | | |
|---|---|---|---|---|---|
| | | Gboko | Hospital | | Initiative Nigeria/FHI |
| | Makurdi | General Hospital, North Bank | Secondary Hospital | Urban | International Center for AIDS Care and Treatment Programs |
| Kaduna | Jama'a | General Hospital, Kafanchan | Secondary Hospital | Urban | International Center for AIDS Care and Treatment Programs |
| Kano | Kano Municipal | Hasiya Bayero Pediatric Hospital | Secondary Hospital | Urban | Global HIV/AIDS Initiative Nigeria/FHI |
| | Gezewa | General Hospital, Gezewa | Secondary Hospital | Rural | Institute of Human Virology Nigeria |
| Lagos | Mushin | Lagos University Teaching Hospital (LUTH), Idi Araba-Lagos | Tertiary Hospital | Urban | AIDS Prevention Initiative in Nigeria /Havard |
| | Lagos Island | Massey Children's Hospital, Isale Ganga-Lagos | Tertiary Hospital | Urban | Global HIV/AIDS Initiative Nigeria/FHI |
| | Ikeja | Lagos State University Teaching Hospital (LASUTH), Ikeja | Tertiary Hospital | Urban | Institute of Human Virology Nigeria |
| FCT-Abuja | Gwagwalad a | University of Abuja Teaching Hospital (UATH), Gwagwalada | Tertiary Hospital | Peri-urban | Institute of Human Virology Nigeria |
| | Bwari | St. Vincent's Daughters of Charity Hospital, Kubwa | Secondary Hospital | Peri-urban | AIDSRelief |
| | Amac | General Hospital, Wuse | Secondary Hospital | Urban | Global HIV/AIDS Initiative Nigeria/FHI |
| | | Maitama District Hospital, Maitama | Secondary Hospital | Urban | Global HIV/AIDS Initiative Nigeria/FHI |
| | | Asokoro District Hospital, Asokoro | Secondary Hospital | Urban | Institute of Human Virology Nigeria |

# APPENDIX D

# BASELINE CHARACTERISTICS BY SITE

| | | | | | | | | | | | | | *Currently on…* | | | | | |
|---|---|---|---|---|---|---|---|---|---|---|---|---|---|---|---|---|---|---|
| | | | **Age at initiation in months** | | **Initial CD4 count** | | **Initial CD4 percentage** | | **Severe immune-suppression** | | **Weight for age (z-score)** | | **AZT/3TC/NVP** | | **Regimens containing EFV (%)** | | **Regimens containing LPV/r (%)** | |
| **Site Name** | **District** | **Total N** | **N** | **Mean (SD)** | **N** | **Mean (SD)** | **N** | **Mean (SD)** | **N** | **%** | **N** | **Mean (SD)** | **N** | **%** | **N** | **%** | **N** | **%** |
| Lagos University Teaching Hospital (LUTH), Idi Araba-Lagos | LAGOS | 85 | 85 | 48.07 (43.96) | 73 | 853.12 (743.78) | 21 | 26.89 (20.43) | 25 | 33.78 | 77 | -1.88 (1.85) | 74 | 87.06 | 6 | 7.06 | 2 | 2.35 |
| Massey Children's Hospital, Isale Ganga-Lagos | LAGOS | 90 | 90 | 58.43 (46.45) | 85 | 787.55 (641.27) | 45 | 28.02 (57.88) | 47 | 55.29 | 85 | -1.41 (3.7) | 50 | 55.56 | 25 | 27.78 | 0 | 0 |
| Lagos State University Teaching Hospital (LASUTH), Ikeja | LAGOS | 83 | 83 | 55.77 (41.85) | 76 | 894.5 (786.98) | 53 | 18.35 (12.37) | 36 | 45.57 | 81 | -1.53 (1.93) | 74 | 89.16 | 1 | 1.2 | 3 | 3.61 |

| | | | | | | | | | | | | | | | | | | |
|---|---|---|---|---|---|---|---|---|---|---|---|---|---|---|---|---|---|---|
| University of Abuja Teaching Hospital (UATH), Gwagwalada | FCT-ABUJA | 84 | 81 | 67.65 (40.89) | 82 | 766.51 (625.89) | 34 | 17.99 (11.64) | 32 | 40 | 80 | -1.44 (3.58) | 71 | 84.52 | 6 | 7.14 | 1 | 1.19 |
| St. Vincent's Daughters of Charity Hospital, Kubwa | FCT-ABUJA | 68 | 68 | 58.98 (47.19) | 66 | 829.12 (573.02) | 59 | 20.68 (13.82) | 29 | 44.62 | 64 | -1.42 (1.83) | 42 | 61.76 | 7 | 10.29 | 9 | 13.24 |
| Annunciation Specialist Hospital, Eneme | ENUGU | 69 | 69 | 79.95 (55.74) | 48 | 512.56 (421.89) | 18 | 18.34 (13.79) | 31 | 47.69 | 56 | -0.87 (4.77) | 35 | 50.72 | 12 | 17.39 | 17 | 24.64 |
| University of Nigeria Teaching Hospital, Ozalla | ENUGU | 73 | 72 | 62.39 (36.47) | 72 | 748.36 (552.51) | 73 | 22.06 (13.26) | 35 | 48.61 | 71 | 0.87 (2.98) | 47 | 64.38 | 5 | 6.85 | 19 | 26.03 |
| Udi District Hospital, Udi | ENUGU | 47 | 46 | 72.29 (53.61) | 28 | 384 (406.34) | 11 | 16.32 (7.08) | 22 | 57.89 | 42 | -2.49 (3.47) | 39 | 82.98 | 3 | 6.38 | 0 | 0 |
| General Hospital Calabar | CROSS RIVER | 71 | 71 | 61.55 (50.12) | 68 | 720.78 (613.79) | 4 | 12.4 (4.75) | 25 | 36.76 | 64 | -0.82 (2.47) | 62 | 87.32 | 6 | 8.45 | 0 | 0 |
| General Hospital, Ogoja | CROSS RIVER | 72 | 72 | 49.51 (41.45) | 70 | 824.1 (561.12) | 29 | 17.68 (10.36) | 31 | 43.66 | 66 | -0.69 (4.76) | 67 | 93.06 | 2 | 2.78 | 1 | 1.39 |
| University of Uyo Teaching Hospital, Uyo | AKWA IBOM | 72 | 72 | 48.75 (43.49) | 64 | 634.75 (473.48) | 7 | 16.27 (9.52) | 29 | 45.31 | 66 | -1.25 (3.2) | 70 | 97.22 | 2 | 2.78 | 0 | 0 |
| Hsiya Bayero Children Hospital | KANO | 80 | 80 | 55.56 (46.51) | 72 | 674.51 (566.05) | 40 | 14.61 (6.98) | 43 | 58.11 | 76 | -2.5 (5.11) | 68 | 85 | 1 | 1.25 | 0 | 0 |

| | | | | | | | | | | | | | | | | | | |
|---|---|---|---|---|---|---|---|---|---|---|---|---|---|---|---|---|---|---|
| General Hospital, Gezewa | KANO | 3 | 3 | 87.47 (90) | 3 | 237.67 (118.51) | 1 | 25 (n/a) | 1 | 33.33 | 2 | 0.57 (3.2) | 3 | 100 | 0 | 0 | 0 | 0 |
| General Hospital, Kafanchan | KADUNA | 70 | 70 | 65.43 (46.84) | 68 | 720.69 (609.97) | 2 | 38.05 (23.55) | 27 | 39.71 | 68 | 0.23 (4.53) | 67 | 95.71 | 0 | 0 | 0 | 0 |
| State Specialist Hospital, Jalingo | TARABA | 73 | 73 | 72.07 (67.47) | 44 | 566.84 (1045.23) | 28 | 21.4 (11.31) | 23 | 34.85 | 59 | -2 (2.81) | 59 | 80.82 | 6 | 8.22 | 0 | 0 |
| General Hospital, Bida | NIGER | 42 | 42 | 91.78 (56.03) | 42 | 352.17 (362.86) | 0 | | 20 | 47.62 | 37 | -2.43 (4.49) | 42 | 100 | 0 | 0 | 0 | 0 |
| General Hospital, Lapai | NIGER | 34 | 34 | 93.45 (64.4) | 32 | 407.72 (384.35) | 0 | | 11 | 34.38 | 27 | -1.85 (4.64) | 27 | 79.41 | 0 | 0 | 0 | 0 |
| NSKT Hospital, Zaki Biam | BENUE | 49 | 49 | 135.57 (79.08) | 49 | 405.22 (383.54) | 0 | | 18 | 36.73 | 28 | -0.85 (2.29) | 37 | 75.51 | 1 | 2.04 | 0 | 0 |
| General Hospital, Gboko | BENUE | 71 | 71 | 73.33 (55.26) | 43 | 266.05 (253.39) | 30 | 14.31 (10.57) | 35 | 49.3 | 65 | -1.57 (3.29) | 67 | 94.37 | 1 | 1.41 | 0 | 0 |
| General Hospital, North Bank | BENUE | 70 | 70 | 55.92 (35.62) | 69 | 555.06 (424.58) | 3 | 19.27 (12.41) | 30 | 43.48 | 70 | 1.98 (8.04) | 63 | 90 | 5 | 7.14 | 0 | 0 |
| Maitama District Hospital, Maitama | FCT-ABUJA | 85 | 85 | 52.34 (40.78) | 84 | 632.63 (502.56) | 29 | 19.43 (12.46) | 54 | 64.29 | 84 | -1.37 (2.84) | 73 | 85.88 | 12 | 14.12 | 0 | 0 |

| | | | | | | | | | | | | | | | | | | |
|---|---|---|---|---|---|---|---|---|---|---|---|---|---|---|---|---|---|---|
| General Hospital, Wuse | FCT-ABUJA | 79 | 79 | 62.65 (50.73) | 75 | 674.16 (556.86) | 23 | 16.86 (13.29) | 29 | 37.66 | 71 | -1.71 (2.87) | 60 | 75.95 | 2 | 2.53 | 1 | 1.27 |
| Asokoro District Hospital, Asokoro | FCT-ABUJA | 46 | 46 | 50.28 (39.49) | 44 | 834.98 (631.04) | 2 | 12.5 (3.54) | 20 | 45.45 | 43 | 0.76 (5.82) | 39 | 84.78 | 7 | 15.22 | 0 | 0 |

For more information, please visit aidstar-one.com.

**AIDSTAR-One**
John Snow, Inc.
1616 Fort Myer Drive, 16th Floor
Arlington, VA 22209 USA
Phone: 703-528-7474
Fax: 703-528-7480
Email: info@aidstar-one.com
Internet: aidstar-one.com
